# DNA repair glycosylase hNEIL1 triages damaged bases via competing interaction modes

**DOI:** 10.1038/s41467-021-24431-y

**Published:** 2021-07-05

**Authors:** Menghao Liu, Jun Zhang, Chenxu Zhu, Xiaoxue Zhang, Weide Xiao, Yongchang Yan, Lulu Liu, Hu Zeng, Yi Qin Gao, Chengqi Yi

**Affiliations:** 1 Peking-Tsinghua Center for Life Sciences, Peking University, Beijing, China; 2 Academy for Advanced Interdisciplinary Studies, Peking University, Beijing, China; 3 Beijing National Laboratory for Molecular Sciences, College of Chemistry and Molecular Engineering, Peking University, Beijing, China; 4 Institute of Systems and Physical Biology, Shenzhen Bay Laboratory, Shenzhen, China; 5 State Key Laboratory of Protein and Plant Gene Research, School of Life Sciences, Peking University, Beijing, China; 6 Beijing Advanced Innovation Center for Genomics, Peking University, Beijing, China; 7 Biomedical Pioneering Innovation Center, Peking University, Beijing, China; 8 Department of Chemical Biology and Synthetic and Functional Biomolecules Center, College of Chemistry and Molecular Engineering, Peking University, Beijing, China

**Keywords:** Enzyme mechanisms, Base excision repair, X-ray crystallography, Molecular dynamics

## Abstract

DNA glycosylases must distinguish the sparse damaged sites from the vast expanse of normal DNA bases. However, our understanding of the nature of nucleobase interrogation is still limited. Here, we show that hNEIL1 (human endonuclease VIII-like 1) captures base lesions via two competing states of interaction: an activated state that commits catalysis and base excision repair, and a quarantine state that temporarily separates and protects the flipped base via auto-inhibition. The relative dominance of the two states depends on key residues of hNEIL1 and chemical properties (e.g. aromaticity and hydrophilicity) of flipped bases. Such a DNA repair mechanism allows hNEIL1 to recognize a broad spectrum of DNA damage while keeps potential gratuitous repair in check. We further reveal the molecular basis of hNEIL1 activity regulation mediated by post-transcriptional modifications and provide an example of how exquisite structural dynamics serves for orchestrated enzyme functions.

## Abstract (web summary)

hNEIL1 (human endonuclease VIII-like 1) is a broadly specific DNA glycosylase for base excision repair. Here, the authors show that hNEIL1 can assume activated or triage conformations: the structural basis for the mechanism that enables broad specificity and reduces futile repair of normal bases.

## Introduction

The genome integrity of living organisms is constantly subject to a wide range of threats, such as metabolic reactive oxygen species (ROS) and external ionizing irradiations, which inevitably result in various alternations to the chemical structures of canonical nucleobases^1^. These damaged bases are mutagenic and/or cytotoxic to cells and their accumulation can eventually lead to severe diseases including cancer, aging, and neurodegenerative disorders^2,3^. Thus, cells have evolved the base excision repair (BER) pathway to correct the majority of abnormal bases^4^. BER is initiated by lesion-specific DNA glycosylases, which catalyze the cleavage of cognate base substrates and mediate the recruitment of downstream repair machinery to the damage sites^5–7^.

Human endonuclease VIII-like 1 (hNEIL1) is a bifunctional DNA glycosylase of the Fpg/Nei family, which catalyzes the removal of damaged bases and subsequent incision of the newly generated abasic sites^8–10^. Unlike traditional enzymes which are commonly referred to as highly specific biocatalysts, one special feature for hNEIL1 is its unique capability to recognize a wide range of substrates, including formamidopyrimidines, hydantoin lesions, oxidized, and dihydro-modified pyrimidine bases^11–13^, derived from all four canonical bases (Supplementary Fig. 1). Interestingly, there is an editing event occurring on hNEIL1 pre-mRNA which leads to a K to R recoding at residue 242, and these two forms of hNEIL1 enzyme (K242 and R242 for the unedited and edited form, respectively) are shown to have different lesion specificity and glycosylase activity^14^. Because hNEIL1 is increased during the S phase of the cell cycle and can remove base lesions in ssDNA, bubble, and forked structures^15,16^, it has been proposed that hNEIL1 is involved in the process of replication-associated DNA repair^17–19^. Hence, hNEIL1 is an essential enzyme for guarding the genome stability in mammalian cells. In line with this, hNEIL1 abnormality has been related to metabolic syndrome, immunity disorders, and carcinogenesis^20–24^. In addition, emerging kinds of literature have evidenced that hNEIL1 may have a regulatory role in the active DNA demethylation pathway^25–27^.

Many DNA lesions differ from normal DNA by only one or a few atoms; thus, repair enzymes face the daunting task of distinguishing their cognate substrates from the vast excess of normal bases^28^. While there are specific glycosylases like uracil-DNA glycosylase (UNG)^29,30^, 8-oxoguanine DNA glycosylase 1 (hOGG1)^31^, and alkylpurine glycosylase D (AlkD)^32^, DNA glycosylases (for instance, hNEIL1, AlkA [*E. coli* 3-methyladenine DNA glycosylases]^33^, and AAG [human 3-methyladenine DNA glycosylases]^34^) can also have remarkably broad substrate ranges^28^. Such broad specificity of repair is believed to be at odds with exclusion of normal DNA. In fact, there is evidence that repair enzymes are not infallible: several NER and BER enzymes have been shown to act on normal DNA^35–38^. Because the repair substrate is DNA, the carrier of genetic information, even low levels of mistaken activity could jeopardize the integrity of the genome. However, our understanding of the molecular basis of how broad-specificity repair enzymes, including hNEIL1, minimize such risks, i.e. recognize various substrates while suppressing erroneous cleavage of normal bases, is very limited.

Here, by integrating structural, computational, and biochemical investigations, we show that hNEIL1 uses a triage mechanism to achieve broad specificity and reduce futile repair of normal bases. This mechanism involves two mutually competing interaction states: an activated state that commits catalysis and BER, and a quarantine state that temporarily separates and prevents the flipped base from catalysis via auto-inhibition. The dominant interaction state varies for different types of damage and is largely determined by the chemical and structural properties of the flipped bases. Such a mechanism enables hNEIL1 to process a series of structurally diverse substrates while minimizes the risk of erroneous cleavage of normal bases. The equilibrium between the two states can also be modulated by a naturally existing hNEIL1 protein variant or artificially introduced mutations. Furthermore, based on our understanding of such a mechanism, we can reactivate the catalytically incompetent hNEIL1 mutants via rational design.

## Results

### hNEIL1 uses a tautomerization-dependent mechanism to recognize various substrates

**Fig. 1:**
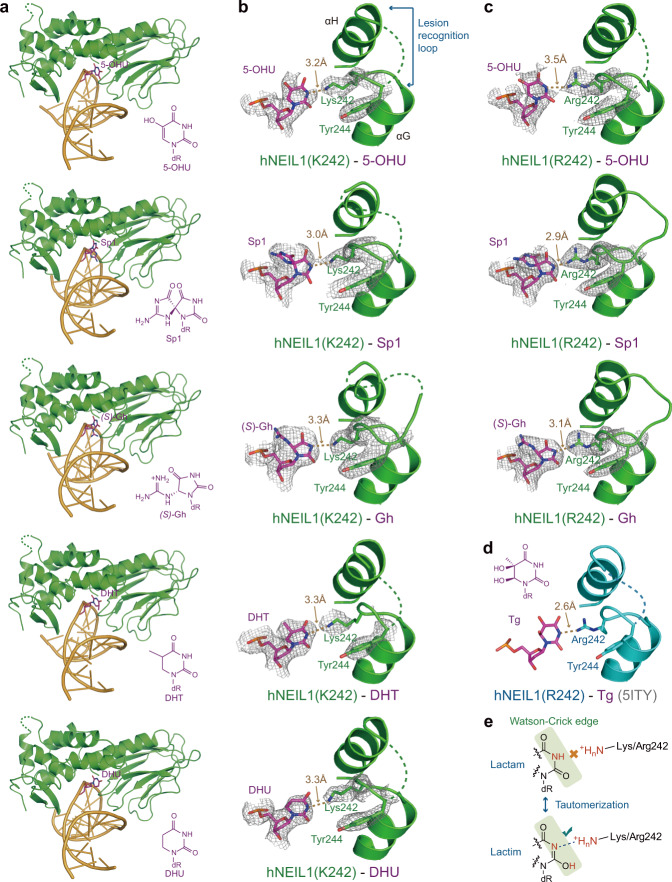
Tautomerization-dependent recognition of diverse substrates by hNEIL1. **a** Overall views of hNEIL1(K242) bound to double-strand DNAs containing a 5-OHU, Sp1, (*S*)-Gh, DHT, or DHU. The chemical structures of individual damaged bases are shown in purple. **b** Zoom-in views of the interaction mode for hNEIL1(K242) in complex with various damaged bases. The lesion recognition loop located between αG and αH is indicated. The 2Fo−Fc simulated-annealing composite omit electron density map is shown in gray mesh and contoured at 1.0*σ* for both damaged bases and key loop residues. The gold dashed line represents hydrogen bond interaction. The dashed curve stands for an unbuildable short region of the lesion recognition loop due to ambiguous electron density. **c** The *242-in* loop conformation was again observed in the crystal structure of a naturally existing variant hNEIL1(R242) in complex with 5-OHU, Sp1, and (*S*)-Gh, respectively. **d** The previously reported hNEIL1(R242)-Tg structure (PDB code: 5ITY) also shows a *242-in* conformation, consistent with the all above described hNEIL1–DNA complexes. **e** The tautomerization-dependent hydrogen bonding between the flipped bases and residue 242 of hNEIL1. The base tautomers are denoted with the lactam (upper) and lactim (lower) forms.

We previously introduced a P2G mutation in the active site of hNEIL1(C∆95) truncation to reduce the catalytic activity, which allowed us to obtain the Tg-containing complex structure^39^. While we also crystallized the complex structures for more lesions in this study, we were unable to obtain intact substrates in the active site of hNEIL1, presumably due to the residual glycosylase activity. Hence, we introduced an extra E3Q mutation to the truncated hNEIL1(C∆95 P2G) (see the “Methods” section) and succeeded in the determination of complex structures containing 5-hydroxyuracil (5-OHU), spiroiminodihydantoin 1 (Sp1), (*S*)*-*guanidinohydentoin (Gh), dihydrothymine (DHT) and dihydrouracil (DHU), respectively (Fig. 1a, Supplementary Fig. 2 and Supplementary Table 1).

Despite the very different chemical structures of these substrates, they are recognized similarly by hNEIL1. The damaged bases are fully extruded from the DNA helix and inserted into a relatively loose binding pocket, which allows the accommodation of substrates with varying sizes and shapes. One edge of the flipped base points towards the protein and forms close contact with the side chain of K242 (Fig. 1b). For a naturally existing hNEIL1 variant (R242)^14^ in complex with dsDNAs containing OHU, Sp1, or Gh, the damaged bases are in close contact with the guanidine group of R242 (Fig. 1c). Such conformation of the lesion recognition loop resembles that in the hNEIL1-Tg structure (Fig. 1d), where the positively charged side chain of residue 242 is contiguous to the solvent-exposed flipped base while the aromatic residue Y244 is laid away from the active site (Supplementary Fig. 3a) (thus called “*242-in*” conformation).

Noteworthy, across all these structures, we observed an unusually short distance (~3.0 Å) between an outwards heterocyclic nitrogen atom of the nucleobases and the terminal nitrogen atom of residue 242. While repulsion is expected for the two groups if both are fully protonated (i.e., an −NH group in the nucleobase and a positively charged side chain of residue 242), we previously showed in the hNEIL1–Tg complex that hNEIL1 utilizes a tautomerization-dependent mechanism to recognize the flipped base^39^. In this mechanism, the base undergoes a keto-enol tautomerization event where the originally protonated heterocyclic nitrogen atom becomes a hydrogen bond acceptor and hence can be hydrogen-bonded to residue 242 (Fig. 1e). Additionally, this tautomerization-dependent hydrogen bond is crucial for the subsequent catalysis by activating the substrate and stabilizing the transition state^39^. Based on the similar overall conformation and detailed atomic interactions, we conclude that such a tautomerization-dependent mechanism may be a general strategy for hNEIL1 to recognize not only Tg but also 5-OHU, Sp1, Gh, DHT, and DHU. Hence, tautomerization can serve as a chemical check to interrogate the tendency to tautomerize for a flipped nucleobase, but not its exact structure.

### A cryptic nucleobase-binding state in hNEIL1

**Fig. 2:**
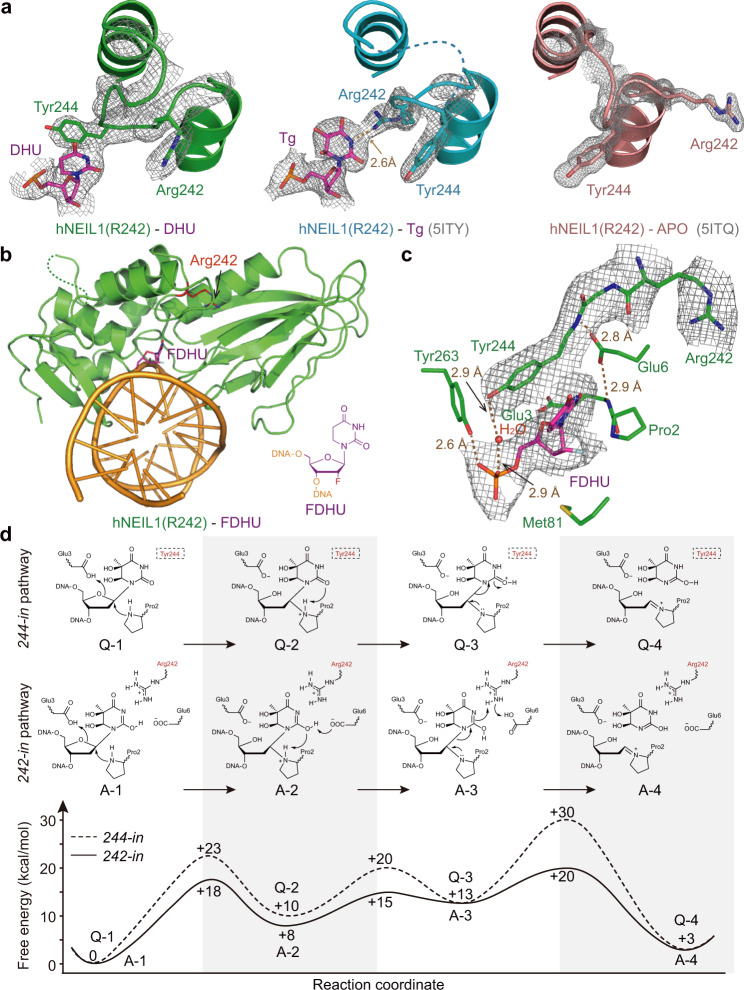
Characterization of a newfound hNEIL1–DNA interaction mode. **a** Crystal structure of hNEIL1(R242) bound to DHU (left) reveals a distinct conformation of lesion recognition loop, compared with the hNEIL1(R242)-Tg structure (middle) or the hNEIL1(R242)-APO structure (right). In the DHU-bound structure, Arg242 occupies the same position which is originally taken by Tyr244 of the Tg-containing structure, while Tyr244 stacks against the flipped DHU base. In addition, Arg242 of the APO structure is placed far away from the damaged bases and fully exposed to the solvent. The gray 2Fo−Fc simulated-annealing composite omit electron density map is contoured at 1.0*σ* for both damaged bases and lesion recognition loop. **b** Overall structure of hNEIL1(R242) bound to dsDNA-containing FDHU. The chemical structure of FDHU deoxynucleotide is shown in purple and residue 242 is colored in red. **c** Zoom-in view of the interactions between hNEIL1(R242) and FDHU. The brown dashed lines represent hydrogen bond interactions and red ball stands for water molecules. **d** QM/MM calculations reveal that the free-energy profile of base cleavage reaction pathway for *244-in* conformation displays an extremely high energy barrier, comparing to a more reasonable *242-in* conformation.

Surprisingly, in the crystal structures of the edited hNEIL1(R242) bound to DHT or DHU, we observed that hNEIL1 can also interact with the substrates in a very different mode (Supplementary Fig. 3b, c). Different from the *242-in* loop conformation described previously, in the structures of hNEIL1(R242)-DHU and hNEIL1(R242)-DHT, R242 residue moves away from the flipped bases while Y244 moves in and stacks against DHU/DHT, burying the nucleobase in a hydrophobic enclosing (Fig. 2a and Supplementary Fig. 3d, e) (termed as the “*244-in*” conformation). Such a conformational change is reminiscent of the “DFG flip” well-known for protein kinases, in which key amino acid residues in a flexible loop can switch their relative positions^40,41^. The *244-in* conformation of lesion recognition loop is also different from that in the hNEIL1 free protein (*apo* conformation), where the loop rotates for ~40° (using Gly240 and Gly249 as turning points) to stay far away (>9 Å) from the position of the flipped base (Fig. 2a). To rule out the possibility that the observed interaction is caused by mutations introduced to hNEIL1 for crystallization experiments, we used an orthogonal approach in which a modified nucleobase substrate is bound by the native protein containing the wild type active site. We synthesized a 2′-fluoro-substituted stable mimic of DHU (FDHU), which allows binding but not catalysis by hNEIL1 (Supplementary Figs. 4 and 5). Indeed, in the hNEIL1(R242)-FDHU structure, residues R242 and Y244 again swaps positions (Fig. 2b, c), recapitulating the observed *244-in* conformation in the hNEIL1(R242)-DHU and hNEIL1(R242)-DHT structures (Supplementary Fig. 3b, c).

Unlike the *242-in* conformation which corresponds to an activated state priming the glycosylase catalysis reaction^39^, what role the *244-in* conformation plays in the function of hNEIL1 remains elusive. Apparently, the hydrogen bond interaction between residue 242 of hNEIL1 and the substrate which is present in the *242-in* conformation is absent in the *244-in* structures, indicating the loss of a key stabilizing factor of binding and catalysis. Thus, we hypothesized that *244-in* conformation is not suitable for executing the glycosylase chemistry. To verify this hypothesis, we performed QM/MM simulations of the ribose-activated catalysis pathway^39,42^ initialized from the hNEIL1(R242)-DHU structure, where the reaction is commonly assumed to be triggered by a nucleophilic attack to C1′ of the ribose by Pro2, followed by the breakage of the glycosidic bond undergoing a highly polarized intermediate (Supplementary Fig. 6). Compared to the *242-in* conformation where a delicate hydrogen bond network is formed in the active site^39^ (Fig. 2d), there lacks catalytically helpful interactions between the substrate and enzyme in the *244-in* conformation, causing several drawbacks hindering the catalysis: (i) the position of the flipped base and ribose becomes more flexible and C1′ can stay farther away from Pro2 (Supplementary Fig. 7), hence the reaction is more difficult to be triggered; (ii) the “proton relay” which facilitates the catalysis in *242-in* conformation is no longer applicable; (iii) the produced highly polarized intermediate cannot be stabilized by a hydrophobic Y244. All these factors contribute to the observed high barrier along the reaction free-energy profile (Fig. 2d and Supplementary Fig. 8). Compared to the *242-in* reaction profile which is in line with other ribose-protonated DNA repair studies^28,42^, the *244-in* pathway could be slowed down by a significantly higher barrier at the glycosylic bond-breaking step (i.e., “Step III” in Fig. 2d) by several orders of magnitude. Thus, the *244-in* conformation is incompetent of glycosylase activity and hence represents a distinct interaction mode with potentially special function.

### A proposed nucleobase recognition model involving two competitive interaction states

**Fig. 3:**
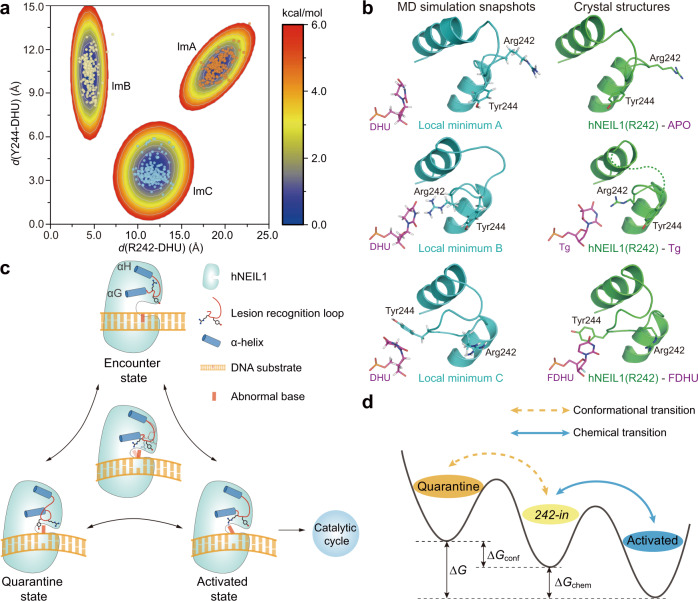
A proposed nucleobase-recognition model of hNEIL1 by computational and structural evidences. **a** Adaptive MD simulations reveal the existence of three conformational states of hNEIL1 when interacting with the substrate DNA (DHU) with relatively enriched population. lmA local minimum A, lmB local minimum B, lmC local minimum C. The dots superimposed are randomly selected MD configurations from each metastable conformation and colored accordingly. **b** The loop conformations of snapshots from adaptive molecular dynamics simulations are in good coincidence with those observed in the crystal structures. **c** Substrate-recognition model of hNEIL1. Upon encountering the DNA substrates, the lesion recognition loop of hNEIL1 shifts towards the flipped bases from the originally preferred *apo* conformation (Encounter state). Once the tautomerization-dependent hydrogen bond interaction between damaged base and residue 242 is established, the substrate would be activated (Activated state) and permitted to enter the catalysis cycle. Alternatively, if the base does not pass the chemical check, the loop would transit to a *244-in* conformation (Quarantine state), temporally restricting the nucleobase from catalysis via competing with the activated state. **d** The relative dominance of the quarantine state and activated state depends on the tug of war between their thermodynamic stability. Δ*G* denotes free energy difference quantifying the relative stability of the activated state over the quarantine state. Δ*G* can be decomposed into two terms, Δ*G*_conf_ (Supplementary Fig. 9) and Δ*G*_chem_, respectively. The gold dashed line represents a process involving conformational transition and the blue solid line means a chemical transition.

One conformation was captured for one particular combination of hNEIL1 protein and base lesion during our crystallographic experiments; and in total, we observed three different conformations for the GS-rich, flexible lesion recognition loop. Whether or not these different conformations could all be adopted by hNEIL1 to recognize a particular substrate is unknown. To fully explore possible metastable protein conformations (as observed in crystals) during hNEIL1–DNA recognition, we performed adaptive MD simulations to explore possible metastable loop conformations in hNEIL1–DNA complexes. The adaptive MD helped explore the loop conformations by interpolating between different crystal structures. We can then identify the metastable conformations by relaxing the perturbed structures generated during the adaptive MD (Supplementary Fig. 9 and Supplementary Table 2). Specifically, inspecting the obtained simulation samples with respect to the relative positions between R242/Y244 and the flipped base, we identified three conformational zones with relatively high population and stability^43^, indicating the existence of (at least) three metastable conformations of the lesion recognition loop upon interacting with DNA (Fig. 3a). Intriguingly, we found that the loop conformations in these three local minima (A, B, and C, respectively) coincide with those observed in three types (i.e., *apo*, *242-in,* and *244-in*, respectively) of crystal structures of hNEIL1 (Fig. 3b). Specifically, in local minimum A, the loop conformation resembles the *apo* form which is experimentally observed for the free protein. Local minimum B consists of *242-in* loop conformation, very similar to the “activated state” defined above, except that the flipped base is not tautomerized. Local minimum C corresponds to the *244-in* conformation that was found in the crystal structure of hNEIL1 bound to DHU/FDHU and DHT. Therefore, MD simulations indicate the co-existence of multiple states for hNEIL1 to interact with a particular nucleobase, among which only the relatively stable one is captured in the crystal structure.

Thus, both our structural and computational observations lead to a substrate recognition model involving multiple interaction states (Fig. 3c and Supplementary Fig. 10). First, the free protein favors a state which has the *apo* loop conformation; hNEIL1 may encounter the DNA in this form (“encounter state”). Once bound to DNA, the recognition loop is shifted away from the *apo* conformation, with the loop moving towards the extruded nucleobase. Along this trajectory, the lesion recognition loop can adjust to a *242-in* conformation, where hNEIL1 exploits a tautomerization-dependent interrogation mechanism attempting to establish hydrogen bond(s) with the flipped base. The activated state arrives if the chemical check is passed, i.e., the key hydrogen bond is established, and the substrate is activated and permitted to enter the catalytic cycle for excision. Alternatively, the lesion recognition loop may also bypass the activated state and transit to the *244-in* conformation. In contrast to the *242-in* conformation, the *244-in* conformation is catalytically inactive and functionally protects the nucleobase from cleavage, in competition with the activated state. The relative dominance of the two interaction states depends on their thermodynamic stability, which could be quantified by their free energy difference (Fig. 3d).

According to the Michaelis–Menten theory, the enzyme–substrate complex (denoted by “ES”) reaches a binding equilibrium prior to the rate-determining catalysis step, so the overall reaction rate *r* can be described by the product of the catalysis rate constant and the total concentration of the equilibrium ES complex, [ES]. However, in hNEIL1, the existence of a competing catalysis-inactive state leads to an auto-inhibitory mechanism, that is, only a fraction of the ES complex in the activated state [ES]_A_ can go through the subsequent catalysis. Given that [ES]_A_ can be approximated using the free energy difference Δ*G* between the two competing states, we arrived at the following catalysis rate equation:

1 $${r}_{{\rm{hNEIL}}1}={k}_{{\rm{cat}}}{\left[{\rm{ES}}\right]}_{{\rm{A}}}\approx \frac{{k}_{{\rm{cat}}}}{1+{\rm{exp }}\left(\frac{\triangle G}{{RT}}\right)}\left[{\rm{ES}}\right]={\widetilde{k}}_{{\rm{cat}}}\left[{\rm{ES}}\right]$$

2 $$\triangle G=\triangle {G}_{{\rm{conf}}}+\triangle {G}_{{\rm{chem}}}$$

where Δ*G* determines the equilibrium population of the protein–DNA complex in the activated state, and *k*_cat_ is the rate for the glycosylase reaction; Δ*G*_conf_ accounts for the conformational transition of the recognition loop from *244-in* to *242-in* (Supplementary Fig. 9), while Δ*G*_chem_ corresponds to the tautomerization event (Fig. 3d). Note that the equilibrium between the two competing states will influence the overall reaction rate by changing the apparent rate constant ${\widetilde{k}}_{{\rm{cat}}}$. Consequently, hNEIL1 can achieve additional substrate discrimination through the thermodynamic competition between the two competing states, which bears significant impact on the overall rate of the catalysis. If the catalytically inactive state dominates over the activated state (Δ*G* is positive), a smaller initial rate for the catalysis is expected. Therefore, we refer the catalytically inactive state as a “quarantine state”.

### Rational manipulation of hNEIL1

**Fig. 4:**
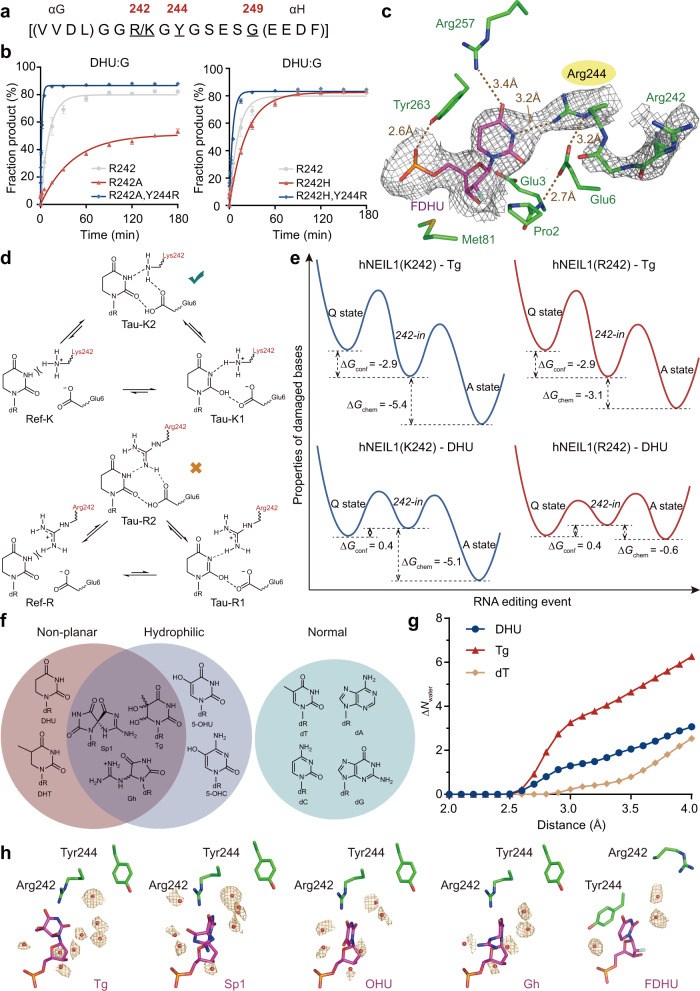
Both key loop residues and base per se contribute to the state transition. **a** The amino acid sequence of lesion recognition loop. Three key residues at position 242, 244 and 249 are indicated. **b** Biochemical assays show that hNEIL1(Y244R) is capable of rescuing the glycosylase activities of 242 mutants on DHU. Values represent mean ± s.e.m. (*n* = 3). See Supplementary Table 3 for calculated rate constants. **c** Zoom-in view of the interactions between FDHU and hNEIL1(Y244R). The simulated-annealing 2Fo−Fc composite omit electron density map is shown in gray mesh and contoured at 1.0*σ*. **d** An additional stable protonation state (“Tau-K2”) can be stabilized by Lys242, but bot by Arg242, to help retain the flipped base in the activated state (illustrated with DHU here). **e** The free energy profile of state transition for hNEIL1(K242) and hNEIL1(R242) bound to duplex DNA containing DHU or Tg. The calculated Δ*G*_conf_ and Δ*G*_chem_ values are determined in kcal/mol. Q state denotes quarantine state and A state stands for activated state. **f** The flipped nucleobases can be categorized by their chemical properties including non-planarity and hydrophilicity. Loss of aromaticity and increase of hydrophilicity of bases would facilitate the formation of a relatively stable activated state. **g** The number of desolvated water molecules (Δ*N*_water_) when the loop transits from *242-in* to *244-in* conformation as a function of the distance from the base computed by MD simulations. **h** The observed solvent environments in the vicinity of the flipped bases within the crystal structures. Water molecules within 5 Å distance of the damaged base are shown here. The annealed difference Fo−Fc electron density map is shown in orange mesh and contoured at 2.5*σ*.

According to the above model, we may swap the preferred interaction states between hNEIL1 and particular substrates, if we are able to inverse the relative dominance of the activated and quarantine states. In light of the observation that different hNEIL1–DNA interaction modes are largely resulted from the conformation changes of the lesion recognition loop, we chose to modify the potential key amino acids on this loop (Fig. 4a). In order to destabilize the quarantine state, we rationally designed hNEIL1(R242, G249P) and hNEIL1(R242, Y244H) to disrupt the flexibility of the loop or the aromatic stacking interactions respectively. Instead of the quarantine state originally observed in hNEIL1(R242)–FDHU structure, we found that both two mutants switched to the activated state as expected (Supplementary Fig. 11a–e).

We then attempted to engineer the enzyme activity via manipulation of the activated state. According to our model, the hydrogen bond(s) between residue 242 and the flipped base is crucial for recognition and catalysis, and disruption of this interaction would destabilize the activated state. As expected, hNEIL1(R242A) or hNEIL1(R242H) demonstrate significantly reduced biochemical activity (Fig. 4b and Supplementary Fig. 11f). As a further test of the proposed mechanism, we also sought to “rescue” the activated state by mutating the lesion-recognition loop. Because a nearby positively charged amino acid side chain is important for catalysis, we attempted to introduce a charged residue in close proximity to the flipped base. Considering that Y244 is able to closely contact the base in the quarantine state, we envisioned that replacing Y244 with a positively charged residue might re-establish an active-state-like interaction between the mutated residue 244 and the flipped base, thus possibly rescuing the catalysis activity. To our delight, introduction of Y244R to hNEIL1(R242A) or hNEIL1(R242H) mutant resulted in both mutants to restore their glycosylase activity; in fact, we even found comparable or higher activity for hNEIL1(R242A, Y244R) and hNEIL1(R242H, Y244R) than the wild-type hNEIL1 for DHU and Tg (Fig. 4b, Supplementary Fig. 11f and Supplementary Table 3). We further solved the crystal structure of hNEIL1(Y244R) bound to FDHU (Fig. 4c), and found that although the loop remains at the *244-in* conformation, Arg244 forms a key hydrogen bond to the flipped base, recapitulating the key interaction in the activated state rather than the quarantine state.

On top of the artificially designed mutations above, the naturally existing hNEIL1 variant, caused by an RNA-editing event at position 242^14^, provides a “native” example of perturbation to the enzyme–substrate interaction. Particularly, the chemical property of 242 side chain can directly influence the “chemical check” (Fig. 4d). By undergoing a base–acid equilibrium with the adjacent Glu6, K242 can interact with the flipped base as a hydrogen bond acceptor (denoted as “Tau-K2”), forgoing the pre-requisite of the base-tautomerization (“Tau-K1”). Through QM/MM calculations we found that, due to the moderate basicity of K242 (pKa~10)^44^, “Tau-K2” is indeed a stable configuration of the activated state in addition to the base-tautomerized “Tau-K1”. In contrast, R242 can hardly utilize this alternative chemistry because the guanidine group exhibits strong basicity^44,45^, and no stable configuration of the speculative “Tau-R2” form can be obtained in simulations (Fig. 4d). Accordingly, K242 is more likely to retain the base in the activated state compared to R242, or in other words, the activated state is more favored by the genomically encoded hNEIL1(K242) compared to its RNA-edited counterpart, which is supported by our QM/MM calculations (Fig. 4e and Supplementary Table 4). Thus, although K and R are commonly regarded as similar basic residues, our findings reveal that RNA editing on hNEIL1 can lead to different biochemical outcomes. Indeed, these two forms of hNEIL1 have been shown to have different glycosylase activities where the unedited hNEIL1(K242) is faster than edited hNEIL1(R242) on several substrates^14,39^.

### Molecular basis of substrate recognition

Are the amino acid residues on the key lesion recognition loop fully responsible for the stability of the enzyme–substrate interaction? The fact that different recognition states were crystallographically captured for the same edited hNEIL1(R242) variant bound to different base lesions suggests that the flipped nucleobase per se also contributes to the stability of the interaction states (Supplementary Table 5). hNEIL1 repairs a diverse array of base damage, whose aromaticity and hydrophilicity differ greatly. Oxidized substrates of hNEIL1 are often bulky and contain non-planar structures; this disrupted aromaticity is expected to diminish the π–π stacking with Tyr244 and hence destabilize the quarantine state (Fig. 4f and Supplementary Fig. 12). In addition, oxidation of DNA bases often increases their hydrophilicity. Transition from the activated state to the quarantine state requires de-solvation of the flipped nucleobase; therefore, the more hydrophilic the base is, the higher the energetic penalty would be. Indeed, we observed an increased number of solvated water molecules for hydrophilic bases like Tg compared to normal dT in our all-atom MD simulations (Fig. 4g). Moreover, it is expected that the bases with less solvent interactions (e.g. DHU) would favor the formation of a stable quarantine state compared with the hydrophilic bases (Sp1, 5-OHU, Gh, and Tg). Consistent with this, at a comparable resolution (around 2.42–2.60 Å) of crystal structures containing five different types of lesions, we found quite different solvation environments for the flipped bases (Fig. 4h). Thus, the structural alteration of the damaged bases, including increased hydrophilicity and decreased aromaticity, directly influences hNEIL1–DNA interaction.

We refer such non-covalent interactions (i.e., solvation and hydrophobic interactions) as “structural check”. Such a mechanism is distinct to the “chemical check” we described earlier, in which the side chain of residue 242 helps hNEIL1 interrogate the substrate via tautomerization and specific hydrogen-bonding test. Referring to the previous quantitative model (Eqs. (1) and (2)), Δ*G*_conf_ can be mainly attributed to the structural check, whereas Δ*G*_chem_ is determined by the chemical check. Both checking mechanisms exploit the chemical and physical properties of the flipped bases: bases prone to tautomerization are more likely to pass the chemical check, whereas bases with decreased aromaticity and/or increased hydrophilicity are more likely to pass the structural check. Usually, damaged DNA bases (e.g., oxidized Tg) are more hydrophilic and simultaneously more prone to tautomerization (Supplementary Table 4). Therefore, these two checking mechanisms work synergistically for hNEIL1 to recognize DNA damage.

### Recognition of a normal base by hNEIL1

**Fig. 5:**
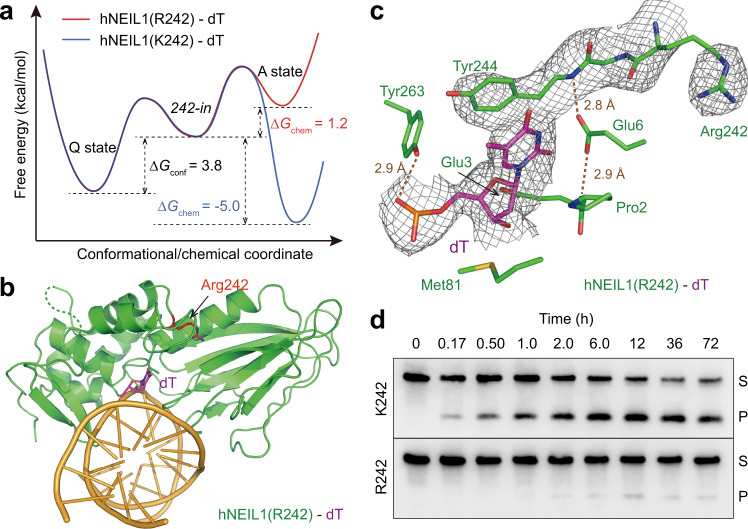
The interrogation of normal base by hNEIL1. **a** The free energy profile of state transition for hNEIL1(R242) and hNEIL1(K242) bound to a mismatched dT. **b** Overall structure of hNEIL1(R242) bound to double-strand DNA containing a T:C mismatch. The flipped dT is colored with purple and Arg242 is shown in red. **c** Zoom-in view of the interactions between dT and hNEIL1(R242). A quarantine state similar to that identified in the crystal structures of hNEIL1(R242) in complex with (F)DHU or DHT is also observed here. The 2Fo−Fc simulated-annealing composite omit electron density map is shown in gray mesh and contoured at 1.0*σ*. **d** An electrophoretic assay shows the glycosylase activities of unedited hNEIL1(K242) and edited hNEIL1(R242) on a T:C mismatch. The experiment was repeated three times independently with similar results. Source data are provided as a Source Data file. Detailed quantitative results can be found in Supplementary Fig. 13.

According to the above substrate-checking mechanism, hNEIL1 interrogates several aspects of the flipped bases, but none of them are specific to any particular substrate. Therefore, hNEIL1 is capable of recognizing and removing a diverse spectrum of DNA damage via some common properties (e.g. non-planarity, hydrophilicity, etc.). However, such a mechanism does not guarantee that an accidentally flipped normal base does not enter the activated state and thus being spuriously removed by hNEIL1. Take a normal dT for example: on the one hand, its intact aromaticity and hydrophobicity is expected to prevent dT from passing the two checks, while on the other hand, the outcome of the competition between the interaction states is also influenced by the protein, including the key 242 residue. To examine how hNEIL1 recognizes dT, we conducted all-atom MD simulations to quantify the thermodynamic stability of the quarantine and activated states of hNEIL1(K242) and (R242) bound to dT, respectively. In the simulated hNEIL1(R242)–dT complex, we observed that the quarantine state is more stable than the activated state and Δ*G* for hNEIL1(R242)–dT is also significantly larger than that of hNEIL1(R242)–Tg (Fig. 5a and Supplementary Table 4), which is consistent with our expectation. We further solved the crystal structure of hNEIL1(R242) bound to dsDNA with a T:C mismatch (Fig. 5b). Indeed, the thymine base is “trapped” in the *244-in* conformation (Fig. 5c), consistent with the quarantine state also observed for hNEIL1(R242)–DHT and hNEIL1(R242)–DHU complexes. These computational and experimental results further support our model that the quarantine state can help hNEIL1 discriminate normal bases from DNA damage.

We next performed simulations for hNEIL1(K242)–dT, which prefers damage recognition using the activated state, to examine whether K242 would differentially influence the recognition of a normal dT. We calculated the relative stability of the two competing interaction states for hNEIL1(K242)–dT. Surprisingly, we found that the two states are of similar stability, hinting that dT is more likely to undergo catalysis in the presence of hNEIL1(K242) (Fig. 5a and Supplementary Table 4). This observation from simulation was further supported by a new crystal structure, where we found that hNEIL1 adopts the *242-in* conformation and that the thymine base is actually removed by hNEIL1(K242) *in crystallo* (Supplementary Fig. 13). Furthermore, our biochemical data demonstrate that over the course of 72 h, hNEIL1(K242) is capable of cleaving a significantly higher portion of dT than the edited form R242 (using a duplex DNA containing a mismatched T:C pair) (Fig. 5d and Supplementary Fig. 13). Taken together, these results show that the genomically encoded K242 is faster in damage repair but more prone to error, while the edited R242 is slower but safer.

## Discussion

hNEIL1 represents an example of broad-specificity DNA glycosylases. A question remains on how a DNA repair enzyme efficiently recognizes so many structurally diverse substrates while remains discriminative towards the normal bases that are in vast excess in the genome. Our study reveals that instead of utilizing a specific pocket to tightly recognize a flipped base via extensive electrostatic and hydrogen bonding interactions, hNEIL1 uses two competing interaction states to achieve substrate recognition. This mechanism is further corroborated by our finding that the relative dominance of the competing interaction states can be manipulated by artificially introduced or naturally existing mutations to the key lesion recognition loop. For instance, based on our mechanistic understanding, we rationally introduced an Arginine mutation to the 244 position of the catalytically incompetent hNEIL1(R242H) and hNEIL1(R242A) mutants, thereby creating new gain-of-function mutants. Thus, such investigation also provides an example that a detailed understanding of reaction mechanisms could lead to the rational engineering of enzymes for tailored biological activities and potentially new-to-nature reactions.

Our crystal structures capture two interchangeable conformations for the lesion recognition loop, where residues 242 and 244 swap their relative positions and switch on and off their catalytic activity. Such conformational flexibility is reminiscent of the well-known “DFG flip”, which connects the catalytically active and inactive conformations of protein kinases^40,41,46^. Based on the DFG flip mechanism, the blockbuster cancer drug imatinib, targeting the “DFG-out” conformation of tyrosine kinases, has been designed for cancer treatment. Given that hNEIL1 abnormality (e.g., single nucleotide polymorphisms) has been identified in metabolic syndrome and several types of cancers^20,22,47,48^, it would be interesting to design small molecules to selectively target different states of hNEIL1–DNA interaction.

Broadly specific glycosylases are thought to be biologically and even evolutionarily beneficial for cells because DNA damage results in a great structural diversity of lesions. Concomitantly, broad specificity enzymes have been shown to act on undamaged, normal DNA, a phenomenon termed as “gratuitous repair”^28^. It is considered to be energetically wasteful and results in increased possibilities of spontaneous mutagenesis^28^. However, accumulated evidence suggests that gratuitous repair is an inevitable price that broad specificity enzymes must pay^35–38^. These findings have led to the notion that DNA repair machineries themselves may be a major source of spontaneous mutagenesis. While the literature has documented such phenomena for a long time, our understanding of how cells minimize gratuitous repair lags behind. For instance, AlkA is known to possess a remarkably indiscriminate active site; hence, once a nucleobase is flipped into the active site, the repair is solely dictated by the reactivity of the N-glycosidic bond of different substrates^35^. In contrast to this catalysis-dependent mechanism, hNEIL1 utilizes a triage mechanism, taking place prior to catalysis, to not only recognize various substrates but also keep potential gratuitous repair activity in check. It remains to be seen to what extent hNEIL1 possesses futile repair activity in vivo. In addition, such pre-catalysis checking mechanism also enabled cells to regulate the repair function of hNEIL1, as demonstrated by the RNA editing event that recodes the key residue at position 242: unedited hNEIL1(K242) is faster but riskier in damage repair, while edited hNEIL1(R242) is slower but safer. In fact, the RNA editing level at this position is dynamically regulated among different tissues (Supplementary Fig. 13d). Therefore, it remains to be seen whether or not this post-transcriptional modification could be exploited by cells as an adaptive response of DNA repair to DNA damage. For instance, in multiple myeloma, only the unedited hNEIL1 can sufficiently relieve the oxidative burden to the genome under elevated ROS conditions^49^; however, this riskier form of hNEIL1 also exposes the cells to potential threats, as a slower growth phenotype has been observed upon over-expression of hNEIL1(K242)^49,50^. Alternatively, it is also conceivable that such a regulatory mechanism could modulate gratuitous repair and hence spontaneous mutagenesis in an evolution-centric view. Collectively, the triage mechanism proposed for a DNA repair glycosylase in this study provides an example to rethink about the specificity of enzymes in maintaining cellular functions.

## Methods

### Oligonucleotides synthesis and purification

5-OHU, DHT, DHU, Tg, and 8-oxo-dG phosphoramidites were purchased from Glen Research. The FDHU phosphoramidite was obtained by chemical synthesis. Damage-containing oligonucleotides were synthesized on an Expedite 8909 DNA synthesizer using standard reagents (Glen Research, Inc.) and deprotected with recommended methods. The resultant oligo DNAs were sequentially purified with GlenPak DNA cartridge and denature PAGE, and desalted on Sep-Pak Cartridge C18 columns (Waters). Complementary strands were purchased from Sangon Biotech (Shanghai). The Gh- and Sp1-containing oligo DNAs were obtained by oxidation of oligomers containing 8-oxoguanine base. All the oligonucleotides were validated by MALDI-TOF-MS. See Supplementary Information for detailed experiment procedures of oligonucleotides preparation and FDHU phosphoramidite synthesis.

### Expression and purification of hNEIL1 proteins

Codon-optimized full-length human *neil1* gene CDS was synthesized by GENEWIZ Inc. and cloned into pET30a (+) with C-terminal 6× His-tag. Truncated hNEIL1 protein was constructed based on this plasmid and point mutations were achieved by Fast Mutagenesis System (TransGen Biotech) (Supplementary Table 6). The plasmid was transformed into BL21 (DE3) (TransGen Biotech) competent cells for protein overexpression. Bacterial cells were grown in LB medium with 50 μg/ml kanamycin monosulfate (AMERSCO) at 37 °C until OD_600_ reached 1.0. Isopropyl β-d-thiogalactoside (IPTG, AMERSCO) was added at a final concentration of 1 mM and cells were then induced for protein expression at 16 °C for 16 h. Collected cells were broken by sonication in lysis buffer (50 mM Na_2_HPO_4_, 300 mM NaCl, 10 mM imidazole, 1 mM DTT, 1 mM PMSF, pH 8.0) and centrifuged at 15,000 rpm for 1 h at 4 °C. The supernatants then passed through a 0.22 μm filter and were ready for subsequent purification. The recombinant proteins were purified at 4 °C with a HisTrap Ni-NTA column (5 ml, GE Healthcare) and Superdex 75 PG chromatography (120 ml, GE Healthcare) using an ÄKTA pure system (GE Healthcare). The equilibration buffer (Buffer A: 50 mM Na_2_HPO_4_, 300 mM NaCl, 10 mM imidazole, pH 8.0) and elution buffer (Buffer B: 50 mM Na_2_HPO_4_, 300 mM NaCl, 500 mM imidazole, pH 8.0) were used for affinity chromatography; the gel filtration buffer (10 mM HEPES, 100 mM NaCl, 1 mM DTT, pH 7.5) was used for size exclusion column. The obtained proteins (about 10–15 mg from 2 L of bacterial cells) were dissolved into gel filtration buffer containing a final concentration of 30% glycerol (v/v) and stored at −80 °C after a fast freezing with nitrogen liquid.

### Crystallization of hNEIL1–dsDNA complexes

Damage-containing oligonucleotides were firstly mixed with the 13-mer complementary strand in a ratio of 1:1.2 and annealed in 1× TN buffer (10 mM Tris, 100 mM NaCl, pH 7.5) to a final concentration of 1 mM. Before the crystallization step, hNEIL1 proteins were first diluted with gel filtration buffer (with no DTT) and concentrated to 8–10 mg/ml to remove the glycerol component by buffer exchange using a 10-kDa ultrafiltration centrifugal tube (Amicon Ultra, Millipore). 24 nmol double-strand DNA was added into 20 nmol hNEIL1 protein and mixed gently by pipetting to give a final protein concentration of ~6 mg/ml. The mixture was then placed on ice for 30 min to form the protein–DNA complex. hNEIL1(CΔ95 R/K242) proteins were used to co-crystallize with double-strand DNA containing FDHU or dT. For DNA with 5-OHU, Gh, Sp1, DHU or DHT base damage, hNEIL1(CΔ95 P2G, E3Q, R/K242) proteins with a GS linker (203–222: GSSGGG) were used for co-crystallization. Orthorhombic rod-shaped crystals grew in 1–2 weeks at 4 °C, in hanging drops containing 2.0 μl of complex solution and 0.8 μl of reservoir solution (100 mM NaCl, 50 mM MgCl_2_, 100 mM cacodylic acid, pH 6.5, 18% PEG 8 K, and 10 mM spermidine). Crystals were transferred into cryoprotectant solution containing reservoir solution plus 25% glycerol (v/v) for equilibration of 1–2 h and eventually stored in liquid nitrogen for data collection.

### Data collection and structure determination

X-ray diffraction data were collected at the Shanghai Synchrotron Radiation Facility (SSRF) beamlines BL17U, BL18U1, and BL19U1 with Mar CCD^51^. The raw data were processed with HKL-2000/3000^52,53^ and phased by molecular replacement with Phaser using the previously determined hNEIL1–DNA complex structure (PDB code: 5ITY) as a searching model. The resulting initial models were built in COOT^54^ and refined with the program REFMAC5 from CCP4 suite^55^. The data collection and refinement statistics for all the structures are listed in Supplementary Table 1. Simulated-annealing composite omit electron density map^56^ of corresponding structures were generated by phenix^57^ and molecular graphic figures were created with PyMOL (https://www.pymol.org).

### DNA cleavage assays

The DNA glycosylase activity of hNEIL1 proteins was evaluated with single-turnover cleavage experiments. Briefly, the 30-mer damage-containing oligonucleotides were firstly annealed with corresponding complementary strands as described above. Then, 20 nM of substrate DNA was mixed with 200 nM active enzyme at 16 °C in a 140 µl of total volume containing 20 mM Tris–HCl, pH 7.5, 10 mM EDTA, 0.1 mg/ml BSA, and 150 mM NaCl. 10 µl of aliquots were removed from the reaction mixture at different time points (0.083, 0.25, 0.50, 1.0, 2.0, 5.0, 15, 30, 60, 90, 120, 150, and 180 min). In view of a relatively slow cleavage rate of hNEIL1 on dT, the sample collection time window was extended to 72 h at 37 °C for DNA substrate containing a T:C mismatch. The reaction was immediately stopped with 10 µl formamide DNA-loading buffer (98% formamide, 10 mM EDTA, pH 8.0, 0.025% bromophenol blue, 0.025% xylene cyanole) followed by heating to 95 °C for 5 min. Inactivated samples were kept on ice until all samples for the time series were obtained. The reaction products were finally separated by 15% PAGE containing 8 M urea and visualized by ChemiDoc MP imaging system (Bio-Rad). The fraction of product for each time point was calculated by dividing the intensity of the product band by the sum of intensities of both product and substrate bands in each lane. Three independent experiments were performed for all the substrate–enzyme combinations. To calculate the catalytic turnover rate *k*_obs_ (min^−1^), the following nonlinear regression (one-phase association) model was used to fit the curves in GraphPad Prism 7.0: fraction product = *A*(1−exp(−*k*_obs_*t*)), where *A* represents the amplitude, *k*_obs_ is the rate constant, and *t* is the reaction time (in minutes).

### Calculation overview

The all-atom models for 15 different hNEIL1–DNA complexes (Supplementary Table 2) were built on the basis of the crystal structures. In some crystal structures where the loop regions were less resolved, we always consulted the high-resolution crystal structures which exhibit the corresponding conformations when possible. The all-atom complex structures were modeled by AMBER-ff99SB force field^58^ and saturated with explicit water models. Before productive MD simulations, all of the all-atom models first underwent relaxations to reduce and equilibrate the improper local structures. The tautomerization energy of the active pocket (i.e. Δ*G*_chem_) in all the atomistic models and the free energy profile of *244-in* catalysis pathway were achieved using QM/MM simulations, where the semi-empirical SCC-DFTB^59,60^ method was employed to describe the QM-treated regions of the atomistic models. The free energy catalysis pathway was calculated by means of umbrella sampling^61^ along prescribed reaction coordinates following the related studies^39^. Force-field simulations were performed to adaptively sample the metastable conformations of the recognition loop, and to compute the free energy of loop conformational transitions (i.e. Δ*G*_conf_) via enhanced sampling. Besides, brute-force force-field simulations were also conducted to investigate the structural fluctuations of the active pocket (including the Y244-base stacking and P2-base distance, etc.). All the simulations were conducted on AMBER18 molecular simulation package^62^.

### Reporting summary

Further information on research design is available in the Nature Research Reporting Summary linked to this article.

## Supplementary information

Supplementary information

Reporting Summary

## Data Availability

Atomic coordinates and structure factors have been deposited in the Protein Data Bank under accession codes 6LWA, 6LWB, 6LWC, 6LWD, 6LWF, 6LWG, 6LWH, 6LWI, 6LWJ, 6LWK, 6LWL, 6LWM, 6LWN, 6LWO, 6LWP, 6LWQ and 6LWR. Previously reported hNEIL1(R242)-Tg structure and hNEIL1(R242)-APO structure are available from the PDB under accession ID 5ITY and 5ITQ, respectively. The RNA-seq data that support the findings of this study are available in the NCBI Sequence Read Archive under accession number SRP039090. Source data are provided with this paper.

## Source data

source data
