# Supplementary material for: DNA repair glycosylase hNEIL1 triages damaged bases via competing interaction modes
Source: Nat Commun. 2021 Jul 5;12:4108. doi: 10.1038/s41467-021-24431-y (PMC8257757; doi:10.1038/s41467-021-24431-y)
Supplement: Supplementary file 1 — Supplementary information [file 41467_2021_24431_MOESM1_ESM.pdf]

Supplementary Information

DNA repair glycosylase hNEIL1 triages damaged bases via competing interaction modes

Menghao Liu[1,2,*], Jun Zhang[3,4,*], Chenxu Zhu[5,*], Xiaoxue Zhang[1,2], Weide Xiao[5], Yongchang Yan[1,2], Lulu Liu[1,2], Hu Zeng[5], Yi Qin Gao[3,4,6,7,†] and Chengqi Yi[1,5,8,†]

Affiliations:

[1] Peking-Tsinghua Center for Life Sciences, Peking University, Beijing 100871, China.

[2] Academy for Advanced Interdisciplinary Studies, Peking University, Beijing 100871, China.

[3] Beijing National Laboratory for Molecular Sciences, College of Chemistry and Molecular Engineering, Peking University, Beijing 100871, China.

[4] Institute of Systems and Physical Biology, Shenzhen Bay Laboratory, Shenzhen 518055, China.

[5] State Key Laboratory of Protein and Plant Gene Research, School of Life Sciences, Peking University, Beijing 100871, China.

[6] Beijing Advanced Innovation Center for Genomics, Peking University, Beijing 100871, China.

[7] Biomedical Pioneering Innovation Center, Peking University, Beijing 100871, China.

[8] Department of Chemical Biology and Synthetic and Functional Biomolecules Center, College of Chemistry and Molecular Engineering, Peking University, Beijing 100871, China.

[*] These authors contributed equally to this work.

[†] To whom correspondence should be addressed. Email: gaoyq@pku.edu.cn or chengqi.yi@pku.edu.cn.

Table of contents

Experimental Procedures 3
Computational Methods..6
Supplementary Figures..14
Supplementary Figure 1. Chemical structures of DNA base substrates for hNEIL1. 14
Supplementary Figure 2. Chromatographic curves and gel image for protein purification. 15
Supplementary Figure 3. Characterization of hNEIL1-DNA interactions. 16
Supplementary Figure 4. The three-step synthesis route of FDHU phosphoramidite. 18
Supplementary Figure 5. Design of FDHU for the co-crystallization with hNEIL1............19
Supplementary Figure 6. Ribose-protonated catalysis pathway for *244-in* loop conformation. 21
Supplementary Figure 7. The calculated distribution of attacking distance between N atom of Pro2 and C1' atom of the flipped ribose for different loop conformations............22
Supplementary Figure 8. QM/MM umbrella sampling of the *244-in* catalysis pathway. ...23
Supplementary Figure 9. Umbrella sampling of the free energy of the loop conformational transition. 24
Supplementary Figure 10. A diagram illustrating the landscape of free energy distribution for different hNEIL1-DNA interaction states. 25
Supplementary Figure 11. Manipulation of state transition by rationally designed hNEIL1 mutants. 26
Supplementary Figure 12. The stacking features with Tyr244 for different flipped bases. 28
Supplementary Figure 13. The RNA editing event regulates the DNA repair accuracy and efficiency of hNEIL1. 29
Supplementary Tables...30
Supplementary Table 1. X-ray data collection and refinement statistics. 30
Supplementary Table 2. All-atom models for hNEIL1-DNA complexes............................35
Supplementary Table 3. Rate constants (k_{obs}) of base removal by hNEIL1 and its mutants. 35
Supplementary Table 4. The calculated free energy value (in kcal/mol) of state transition for hNEIL1 variants in complex with different nucleobases...36
Supplementary Table 5. The interaction states of hNEIL1 variants bound to different flipped bases observed in crystal structures. 36
Supplementary Table 6. Primers used for the plasmid construction of hNEIL1 mutants. .37
References...38

Experimental Procedures

1. Oligonucleotides synthesis and purification

5-OHU, DHT, DHU, Tg, and 8-oxo-dG phosphoramidites were purchased from Glen Research. The FDHU phosphoramidite was obtained by chemical synthesis. Oligonucleotides (5'- CGT CCA X GTC TAC -3', where X stands for 5-OHU, DHT, DHU, 8-oxo-dG, FDHU or dT) were synthesized on an Expedite 8909 DNA synthesizer using standard reagents (Glen Research, Inc.) and deprotected with recommended methods followed with a GlenPak DNA cartridge purification. The resultant oligo DNAs were further purified with denaturing polyacrylamide gel electrophoresis and desalted on Sep-Pak Cartridge C18 columns (Waters). Eluted oligonucleotides were lyophilized and finally dissolved into ddH_2O. Complementary strand (5'- TAG ACC TGG ACG G -3') was purchased from Sangon Biotech (Shanghai) and purified with urea PAGE followed by an aforementioned desalting step. The 30-mer oligonucleotides with damaged bases (5'-Biotin- TGT TCA TCA TGG GTC Y TCG GTA TAT CCC AT -3', where Y denotes DHU, Tg or normal dT) were synthesized as above and purified with GlenPak DNA cartridge. The 30-mer complementary strands (5'- ATG GGA TAT ACC GAZ GAC CCA TGA TGA ACA -3', where Z stands for A, G or C) were purchased from Sangon Biotech without any further purification. All the oligonucleotides were validated by MALDI-TOF-MS.

2. Generation of Gh- and Sp1- containing oligo DNAs

The Gh- and Sp1- containing oligo DNAs were obtained by oxidation of oligomers containing 8-oxoguanine base referring to a previous study with minor modifications[1]. Briefly, to generate the Gh-containing oligo DNA products, 5 nmol 8-oxo-dG oligomers were firstly added into ddH_2O and incubated at 4 °C for 30 min. Then 90 nmol Na_2IrCl_6 (SIGMA-ALDRICH) were titrated into the sample in a 500 μl final volume followed by 50 minutes of incubation. The reaction was finally quenched with 500 nmol EDTA (pH 8.0). To obtain the Sp1 oligo DNA products, 5 nmol 8-oxo-dG oligomers were dissolved in 75 mM Na_3PO_4 buffer (pH 7.4) and incubated at 45 °C for 30 min. The reactions were started by addition of 90 nmol Na_2IrCl_6 and terminated with 500 nmol EDTA (pH 8.0) after 50 minutes of incubation. Oligo DNA products were finally desalted on Sep-Pak Cartridge C18 columns (Waters) and purified with HPLC. The Gh- and Sp1- containing oligo DNA were validated by MALDI-TOF-MS.

3. Synthesis of FDHU phosphoramidite

(i) General

^{1}H, ^{13}C and ^{31}P NMR spectra were measured on a Bruker AVANCEIII spectrometer at 400 MHz. Mass spectra were performed on a Bruker Apex IV FTMS via ESI ionization. Commercially available reagents, fluorodeoxyuridine (99%) was purchased from HEOWNS, Rhodium (5% on carbon, dry) was purchased from Alfa Aesar, pyridine (99.5%, SuperDry, water≤30 ppm) and dichloromethane (99.9%, SuperDry, with molecular sieves, water≤30 ppm) was purchased from *J&K* Scientific, 4,4'-dimethoxytrityl chloride (≥97.0%) was purchased from SIGMA-ALDRICH, *N*,*N*-diisopropylethylamine (99.5%) and 2-cyanoethyl *N*,*N*-(diisopropylchloro)-phosphoramidite (97%) were purchased from Energy Chemical. The three-step synthesis route of FDHU phosphoramidite is shown with details in Supplementary Fig. 4.

(ii) 2'-deoxy-2'-fluoro-5,6-dihydrouridine

The fluorodeoxyuridine **1** (1.4g, 57 mmol) was dissolved in 10 mL water with several drops of concentrated ammonia. Rhodium on carbon (5 wt %, 342 mg) was added to the solution, and the mixture was stirred in hydrogen atmosphere at room temperature for 24 h[2]. The reaction mixture was then filtered with celite and lyophilized. The residue was purified by silica gel column chromatography with MeOH/CH_2Cl_2 (1:8) to give **2** (980 mg, 71%) as a white solid. ^{1}H NMR (DMSO-d_6, 400 MHz): δ = 10.35 (s, 1H), 5.87 (dd, J = 20.4, 3.2 Hz, 1H), 5.50 (d, J = 6.0 Hz, 1H), 5.06 (dd, J = 4.8, 3.2 Hz, 1H), 4.87-4.95 (m, 1H), 3.96-4.07 (m, 1H), 3.68-3.75 (m, 1H), 3.60-3.68 (dq, J = 12.0, 2.8 Hz, 1H), 3.39-3.52 (m, 2H), 3.12-3.22 (d, J = 5.2 Hz, 1H), 2.51-2.63 (m, 2H) ppm; ^{13}C NMR (DMSO-d_6, 101 MHz): δ = 170.80, 153.29, 92.54 (d, J = 184.8 Hz), 86.82 (d, J = 33.3 Hz), 82.80, 68.94 (d, J = 16.2 Hz), 60.78, 36.80, 31.15 ppm. HR-FTMS (ESI) for $C_9H_{13}FN_2O_5$ (*m*/*z*): calculated: 249.0881 $[M+H]^+$; observed: 249.0873.

(iii) 5'-*O*-(4,4'-Dimethoxytrityl)-2'-deoxy-2'-fluoro-5,6-dihydrouridine

Compound **2** (500 mg, 2.0 mmol) was dissolved in pyridine (10 mL), and 4,4'-dimethoxytrityl chloride (813 mg, 2.4 mmol) was added to the solution. After being stirred overnight at room temperature under a nitrogen atmosphere, the reaction mixture was quenched with MeOH (0.5 mL) and stirred for an additional 10 min[3]. The solution was diluted with EtOAc (150 mL), then washed with water (2×60 mL) and brine (60 mL). Organic phases were dried over anhydrous Na_2SO_4 and concentrated under vacuum. The residue was purified by silica gel chromatography with EtOAc/Petroleum Ether (1:1 to 5:1) containing 1.5% Et_3N to give **3** (880 mg, 80%) as a pale yellow foam. ^{1}H NMR ($CDCl_3$, 400 MHz): δ = 7.98 (s, 1H), 7.38 (d, J = 7.2 Hz, 2H), 7.26-7.31 (m, 6H), 7.19-7.24 (m, 1H), 6.82 (d, J = 8.4 Hz, 4H), 5.98 (dd, J = 19.6 Hz, 2.8, 1H), 5.06 (ddd, J = 56.8, 54.0, 3.2 Hz, 1H), 4.36-4.49 (m, 1H), 3.96-4.00 (m, 1H), 3.78 (s, 6H), 3.60-3.68 (m, 1H), 3.46-3.54 (dd, J =

10.8, 2.8 Hz, 1H), 3.31-3.41 (m, 2H), 2.42-2.62 (m, 3H) ppm; ^{13}C NMR ($CDCl_3$, 101 MHz): δ = 169.43, 158.64, 152.22, 144.48, 135.50, 135.47, 130.09, 128.14, 127.90, 127.06, 113.20, 92.40 (d, J = 185.8 Hz), 87.65 (d, J = 33.3 Hz), 86.70, 81.45, 70.22 (d, J = 17.2 Hz), 62.58, 55.26, 37.94, 30.97 ppm. HR-FTMS (ESI) for $C_{30}H_{31}FN_2O_7$ (*m*/*z*): calculated: 573.2008 $[M+Na]^+$; observed: 573.1996.

(iv) 5'-*O*-(4,4'-Dimethoxytrityl)- 3'-*O*-(2-cyanoethyl-*N,N*-diisopropyl)phosphoramidite-2'-deoxy-2'-fluoro-5,6-dihydrouridine

Compound **3** (250 mg, 0.45 mmol) was dissolved in anhydrous CH_2Cl_2 (2.8 mL), and redistilled *N*,*N*-diisopropylethylamine (473 µl, 2.67 mmol) was added to the solution on an ice bath followed by 2-cyanoethyl *N*,*N*-(diisopropylchloro)-phosphoramidite (300 µl, 1.36 mmol). The reaction mixture was stirred at 0 °C under argon atmosphere for 1 h and transferred to room temperature followed with an additional stirring for 2 h. The reaction was finally quenched with MeOH (1 mL) and 2% $NaHCO_3$ (10 mL), and extracted with EtOAc (2×20 mL). Organic phases were washed with brine (30 mL), dried over anhydrous Na_2SO_4 and concentrated. The residue was purified by silica gel chromatography with EtOAc/Petroleum Ether (1:3) containing 1.5% Et_3N to give **4** (236 mg, 70%) as a white foam. ^{31}P NMR (CD_3CN, 202 MHz): δ = 149.64 and 149.59 ppm. HR-FTMS (ESI) for $C_{39}H_{48}FN_4O_8P$ (*m*/*z*): calculated: 751.3267 $[M+H]^+$; observed: 751.3250.

Computational Methods

1. Setup of all-atom models for hNEIL1-DNA complexes

The crystal structures of hNEIL1-DNA complexes obtained through experiments were used to build the simulation systems. Additionally, for simulations of equilibrium fluctuations of different loop conformers, we established several all-atom structures which were not observed in experiments by “mutating” some residues in the structure.

For specificity, we named each model according to the following format: (corresponding base type)-(loop residues/mutations)-(loop conformation). For example, Tg-R242-242in corresponds to the model in which the damaged base is Tg, and the loop has an arginine resided on 242 position and adopts a *242-in* conformation. For simplicity, we also assigned an index for each model. The models directly built from crystal structures have a “crystal” tag for the origin; whereas those containing mutations were tagged by “mutated” from the corresponding crystal-structure model. We listed all the models and their corresponding indices and origins in Supplementary Table 2. We will refer to these models by their indices hereafter.

Furthermore, in order to build reliable initial structures for our purposes, we adopted multi-references to construct the recognition loop. Particularly, if the electron density of the recognition loop in a crystal structure is poor, we will first confirm its loop conformation to be either apo or *242-in* or *244-in*, using the original crystal structure. We then preserve the well-resolved regions of the loop and the other part including the active pocket, and consult the high-resolution crystal structure which has the same loop conformation for reference to make up the less-resolved or missing parts. Note that although some regions of the flexible loop may suffer low resolution, the positions of the active site, including the R/K242, Y244 and E6 etc., are mostly well-defined in the crystal structures.

It should be noted that the mutated residues which deactivated the catalysis pocket introduced in crystallization experiments were recovered to wild types during the above modeling procedure. The heavy atoms including water molecules with high density in crystal structure were preserved, while those of ions and other agents (e.g., glycerol, Tris) were removed. The hydrogen atoms in the protein and DNA were automatically recovered by tLEAP module (AMBER tools) according to AMBER-ff99SB[4]. Counter ions were added to neutralize the overall system. We then added extra water molecules to the system, ensuring each atom of the protein-DNA complex (and ions) were encompassed by water shell at least 10 Å thick. TIP3P water model[5] was adopted to describe the explicit solvation. Each all-atom model contains about 50,000 atoms. The force-field parameters for the unnatural bases involved in the models (i.e., Tg and DHU) were taken from or prepared as

in reference[6].

It is worth mentioning how we treated the protonation states of residues in the hNEIL1 protein. Since the residue 242 (i.e., R242 or K242) in the recognition loop may be subjected to tautomerization in the *242-in* loop conformation, one may concern whether its protonation state is different from titratable conditions. We examined its environment and found that K/R242 is readily accessible to solvent water molecules in both crystal structures (Fig. 4h) and atomistic simulations (Fig. 4g). Besides, we also observed a salt bridge can be formed between K/R242 with an adjacent Glu6 residue in the active pocket. Therefore, we assumed that K/R242 residue is protonated or charged in the 242-in loop conformation. The H++ server (3.2 version; http://biophysics.cs.vt.edu/H++)[7] also verified our assumption of a charged K/R242 under a neutral pH condition. The protonation states of other residues were also set as suggested by the H++ server.

<u>2. Force-field relaxation of all-atom models of hNEIL1-DNA complexes</u>

Unless specified otherwise, in all the simulations, we treated the simulation box with periodic boundary conditions, and set a 10 Å cutoff for Ewald summation accounting for the electrostatic interactions. All the simulation models were treated by the according force fields.

We first minimized the energy of the initial structures using 5000 steps of steepest gradient descent followed by 5000 steps of conjugate gradient descent. After energy minimization, each of the simulation systems was heated up to 300 K under 1 atm, during which a positional restraint (10.0 kcal·mol·Å^{-2}) over protein and DNA was applied. SHAKE algorithm[8] was adopted to constrain all motions involving hydrogens, and a uniform 2-fs step was used for all force-field MD simulations. The heating process lasted for 0.5 ns, followed by 1 ns equilibration stage, during which the positional restraint was gradually tuned off (reducing 1.0 kcal·mol·Å^{-2} every 100 ps). The final structures reached at the end of the equilibration was then used for initializing all the following simulation studies.

<u>3. Calculation of reaction free energy profile for *244-in* pathway of base excision</u>

(i) Selection of QM-treated region

According to references[6,9], in the ribose-protonated reaction pathway, the reactive sites involving significant electronic structure changes (i.e., bond forming and/or breaking) include Pro2 (the Pro2 is neutral at the N-terminal[6]), Glu3 and the flipped nucleotide. Therefore, we treated Pro2, Glu3 and the flipped Tg (including the ribose and the base) by quantum mechanics (QM). Unlike the *242-in* pathway (Fig. 2d in the main text), the

residues on the recognition loop do not directly participate in the *244-in* catalytic pathway, so we treated the recognition loop exclusively by molecular mechanics (MM). All the other regions in the simulation box (e.g., solvent and protein) were all also treated by MM. The QM/MM calculation of the reaction free-energy profile of the hypothetic *244-in* pathway was based on such partitioning of QM and MM regions.

(ii) Umbrella sampling

We followed the hypothetic reaction pathway as in Fig. 2d in the main text. The reaction coordinates were chosen to account for the bond-forming and bond-breaking in each step, which are shown with details in Supplementary Fig.6.

QM/MM steered MD was first performed to generate a series of structures along each reaction coordinate. The semi-empirical quantum method SCC-DFTB[10,11] with proper dispersion terms was adopted to quantify the potential of the QM-treated region as defined in the above section, provided that it generally ensures satisfying accuracy, performs reliably in polar systems, and is computationally extremely efficient compared to the first-principle methods[12]. For every reaction coordinate, the steered MD lasted for no less than 2000 fs (with 1-fs integration time step), which ensured at least 1500 fs for a change of 1 Å along the reaction coordinate so as to relax the environment, and the steering force ranged from 80 to 200 kcal·mol·$Å^{-2}$. The force constant used in steered MD was used as reference for the following umbrella sampling.

We computed the free energy profile along the *244-in* reaction pathway via umbrella sampling[13]. The umbrella sampling was done using SCC-DFTB/AMBER QM/MM simulations where the QM-treated region was identical to the steered MD simulations. SCC-DFTB was chosen so that the result can be fairly compared with related studies[6]. The harmonic restraint (used to keep the configuration locally fluctuated) was in line with the parameters in the steered MD. Specifically, the force constant of the harmonic potential for Step I and Step III are 200, 80, and 160 kcal·mol·$Å^{-2}$, respectively. We chose these values because the corresponding forces were needed to overcome the reaction barrier(s) during the steered MD. The window was 0.1 Å in width along Step I and Step III, and 0.05 Å for Step II which merely involves proton transfers. The time step was 1 fs, and simulations were executed under NPT ensemble (300 K, 1 atm). For each window, up to 100 ps QM/MM simulation was performed, and the last 80-ps trajectory was collected for data analysis. The weighted histogram analysis method (WHAM)[14] was applied to reconstruct the unbiased free energy profiles for each individual reaction step (Supplementary Fig. 8), where the maxima (transition states) and minima (intermediate and stable states) along each reaction coordinate were extracted and aligned to plot the illustrative overall free-

energy profile throughout the entire reaction (Fig. 2d in the main text).

4. Structural fluctuations of the catalytic pocket and the recognition loop

We performed several force-field MD simulations to study the conformational or structural fluctuations of the hNEIL1-DNA complexes.

(i) Distance between Pro2 and the flipped base

Since the nucleophilic attacking from the nitrogen atom on Pro2 to the C1' atom of the flipped nucleotide is the key step that initializes the enzymatic reaction (Supplementary Fig.6), the distance between these two atoms are thus indicative of the ease of the reaction initialization. Given that the relative positions between the flipped nucleotide and the Pro2 vary in different crystal structures and depend on the loop conformation, we launched force-field simulations using M1 and M7 models (Supplementary Table 2), respectively, to investigate how the loop conformation changes the distance between the two reactive sites. The production MD simulation ran 20 ns for each model, based on which we plotted the distribution of the distance between the N atom of Pro2 and C1' atom of the flipped ribose (Supplementary Fig. 7).

(ii) Configurations of residue 249

We simulated the configurations of the residue 249 in different loop conformers. In total 3 simulations were conducted, using M3, M9 and M13 (Supplementary Table 2), respectively. Specifically, in M3 and M9, residue 249 is a wild-type glycine; whereas in M13, we introduced G249P mutation. M3 and M9 differ with each other in the overall loop conformations: M3 corresponds to a *242-in* loop conformer while M9 to a *244-in* loop. For each system, we ran a force-field MD simulation of 20-ns length. We analyzed the production simulation trajectories and plotted the distribution of the backbone torsions of residue 249 in Supplementary Fig. 11b.

(iii) Stacking between residue 244 and the flipped base

Since the *244-in* loop conformation can be stabilized by the stacking interaction between residue 244 and the flipped base, we performed force-field MD simulations to investigate the stacking interaction. We ran MD simulations for 4 all-atom models in total, including M3, M5, M7 and M14 (Supplementary Table 2). M3, M5 and M7 share a common residue 244, namely, a wild-type tyrosine, but differs with each other in the base type (DHU in M3, dT in M5 and Tg in M7). On the other hand, M14 contains a Y244H mutation (we introduced a neutral Histidine in 244, annotated as "HIE" in AMBER by convention) and the flipped base is DHU. We considered a neutral Y244H mutation hereby because a neutral histidine is supposed to lend itself better for hydrophobic interactions with the base, i.e., pi-

stacking, compared to a highly hydrophilic charged counterpart. Compared to HID, HIE was the more commonly adopted neutral form of histidine in literature, and was recommended as default by the H++ server and the force field. The simulation lasted for 20 ns for each system, and the collected data were used to compute the statistics of the stacking interactions. Specifically, we characterized the strengths of the stacking interaction by two quantities, the overlapping area and the distance between the sidechain of the aromatic sidechain of residue 244 and the base (Supplementary Fig. 11d and Supplementary Fig. 12).

The overlapping area was calculated as follows. We first defined two planes spanned by the aromatic sidechain and the base. We then projected the atomic positions of residue 244 sidechain onto the plane of the base which resulted in a polygon. We also projected the atoms of the flipped base onto the same plane which result in another polygon. The intersections between the projected polygons were computed by PyClipper (open source code from: https://pypi.org/project/pyclipper) and Shapely (open source code from: https://pypi.org/project/Shapely/) python library. Finally, the overlapping area was defined as the encompassed area within the intersections.

Given the defined geometric plane for the base, we computed the distance from the heavy atoms of the aromatic ring of residue 244 to this plane. We averaged all the distances to give the averaged distance (i.e., the distance between aromatic ring of residue 244 and the base) reported in Supplementary Fig. 11d and Supplementary Fig. 12a.

(iv) Solvation of the flipped base

In addition to the computation of stacking strengths, we also analyzed the MD simulation trajectories of M3, M5 and M7 to characterize the solvation states of the flipped base, i.e., DHU, dT and Tg, respectively, where the loop all adopted the *244-in* conformation. To investigate the solvation states of the base under a *242-in* loop conformation, we further conducted 3 more MD simulations for M1, M9 and M11 (Supplementary Table 2) with the same simulation settings as above.

As reported in Fig. 4g in the main text, we defined the distance from the base to a water molecule as the shortest one among all the pairwise distances between the water oxygen and any heavy atom on the base. We then counted the number of water molecules, N_{water}, falling short of a given cutoff as the solvated water number in Fig. 4g.

5. Sampling and free-energy calculation of different loop conformations

(i) Adaptive sampling of the loop conformations

To explore the possible metastable conformations of the recognition loop, we

performed adaptive sampling based on force-field MD simulations. The MD simulations were initialized using M3 model (Supplementary Table 2), where the loop adopts a *244-in* conformation in keeping with the crystal structure. To boost the conformational changes of the loop, we implemented two accelerated sampling techniques. First, we collected the torsional angles (Φ, Ψ) of every residue on the loop (residue 240 to 249, as shown Fig. 4a in the main text) as the collective variables (CVs). Based on these CVs, diffusion map[15] was exploited to determine the "frontier configuration"[16]. After every 100-ps MD simulation, we selected a frontier configuration and re-started the MD simulation thereof.

Second, we guided the simulation by a target potential (which is a function of the CVs). Similar to targeted MD, the target potential takes a harmonic form which has the equilibrium position as a target value of the CVs. We used the backbone torsions in the crystal structure as the target values for the CVs provided that little conformational deviations of the recognition loop residues were observed during relaxations of the atomistic models. We imposed a dynamic schedule over the stiffness (or the force constant) of the harmonic potential: In each 100-ps long MD simulation, the stiffness was gradually tuned up during a warm-up stage in the first 50-ps simulation, then decayed to zero during the remaining 50-ps simulation. Two crystal structures were employed to generate the reference target values: one has a *242-in* loop conformation (crystal structure 6LWG, Supplementary Table 2) and the other has an *apo* loop conformation (crystal structure 5ITQ, Supplementary Table 2), and we launched two independent adaptive sampling MD targeted to these two references. The adaptive sampling was terminated once the final configuration exhibited a CV value agreeing with the target.

The adaptive MD helped explore the loop conformational space by interpolating between different crystal structures. However, since the adaptive MD simulations were off-equilibrium, the generated samples cannot represent the equilibrium distribution (or free energy). Indeed, the structures generated along the adaptive MD were mostly perturbed conformations. As in Markov state models (MSM)[17] and information distilling of metastability (IDM)[18], we relaxed the yielded configurations by shooting short NPT MD simulations (each lasted for 200 ps) thereof to investigate the metastability of the conformations. Finally, we collected these relaxed samples and projected them to the 2D space spanned by the base-Y244 distance and base-R242 distance, and we fitted the density of the projected 2D space via a Gaussian mixture model (implemented by scikit-learn python library[19] where we evoked a non-isotropic non-diagonal covariate Gaussian mixture model) and reported the result in Fig. 3a of the main text.

(ii) Free energy calculation of the loop conformational transition

The relaxed adaptive sampling and metastability analysis can help identify metastable conformations, however, they cannot give the relative stability of different conformers due to their off-equilibrium nature. In order to quantify the free energy difference between different metastable states, we performed force-field umbrella sampling. Firstly, we selected two metastable conformations which we aimed to compute the relative stability, and obtained a 1-dimensional reaction coordinate through linear discriminant analysis (LDA)[20]. The LDA linearly transforms a set of candidate order parameters (we used the base-Y244 distance, base-R242 distance, Y244-F85 distance and R242-F85 distance as the order parameters) into a 1D combined reaction coordinate. We then divided the resulting 1D reaction coordinate into 90 to 100 uniformly spaced bins (each bin corresponds to a "window" for umbrella sampling), and selected the configurations (yielded by the adaptive sampling) which fall into each bin as the initial configuration for the umbrella sampling of the corresponding window (Supplementary Fig. 9).

In umbrella sampling, we applied harmonic potentials over the reaction coordinate with a force constant of 8000 kJ/nm^2 to ensure good overlap of sampling between adjacent windows. The restraints were implemented via PLUMED plugin[21] for AMBER. For each window, 6 ns MD simulations with the corresponding harmonic restraint force were conducted to generate samples where the last 5-ns simulation samples were collected for analysis. WHAM was implemented to recover the free energy profile along the reaction coordinate using the generated samples from the 100 windows. The uncertainties of the resulting free energy profiles were assessed by a Monte Carlo bootstrapping procedure[22].

For DHU as the damaged base (i.e., M3 in Supplementary Table 2), we computed two free energy profiles in total: One for *apo* to *244-in* loop transition, i.e., $dG_{\mathrm{E}\to\mathrm{Q}}$ (Supplementary Fig. 9 and Supplementary Fig. 10), the other for *244-in* to *242-in* loop transition, i.e., dG_{conf} (Supplementary Fig. 9 and Supplementary Table 4). We also computed dG_{conf} for dT and Tg as the flipped base (Supplementary Fig. 9) following the same umbrella sampling protocol and summarized the results in Supplementary Table 4. The initial structures for these two systems were the same as the above obtained DHU models except that DHU was mutated to the corresponding new base (dT or Tg). Note that in all the above-mentioned umbrella sampling and free energy calculations, the residue 242 in the model was exclusively arginine regardless of the identity of the damaged base. As reported in Supplementary Table 4, throughout this paper we assumed that replacement of R242 by K242 would not change dG_{conf}.

(iii) Quantification of the free-energy difference between Q state and A state.

The umbrella sampling gives the dG_{conf} but not dG_{chem} of the chemical check. In

order to compute the relative stability between Q and A state, we also need to account for the tautomerization equilibrium of the active pocket (Fig. 4d). Since different tautomerization states mainly differ with each other in energy while the entropy difference can be neglected, we approximated dG_{chem} with dE_{taut} (the energy difference between different tautomers). We conducted QM/MM to minimize the tautomer of the active pocket and record the energy of the final configuration. The QM-treated region included the sidechain of residue 242 and Glu6 as well as the flipped base (the ribose is excluded). We adopted the same QM method as the QM/MM umbrella sampling of the catalytic reaction pathway (Section 3 above). dG_{chem} was approximated as the energy difference between the canonical form of the active pocket and the most energetically favorable tautomer. Specifically, we found that deprotonation of R242 sidechain was energetically prohibited, thus $dG_{\mathrm{chem}}(\mathrm{R242}) \approx E_{\mathrm{Tau-R1}} - E_{\mathrm{Ref-R}}$ (Fig. 4d in the main text); whereas for K242, Tau-K2 (i.e., a neural Glu6 and K242 in equilibrium; Fig. 4d) was the most stable tautomer $dG_{\mathrm{chem}}(\mathrm{K242}) \approx E_{\mathrm{Tau-K2}} - E_{\mathrm{Ref-K}}$. The overall free energy difference between Q and A states was defined as $dG_{\mathrm{Q}\to\mathrm{A}} = dG_{\mathrm{conf}} + dG_{\mathrm{chem}}$ (Eq. 2 and Supplementary Table 4).

Supplementary Figures

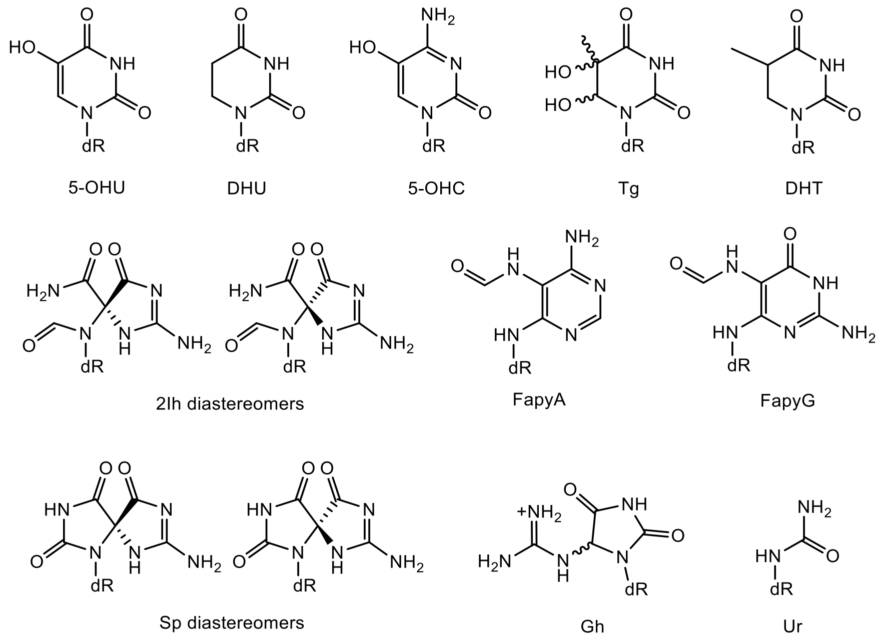

Supplementary Figure 1. Chemical structures of DNA base substrates for hNEIL1. Abbreviations: 5-OHU, 5-hydroxyuracil; DHU, 5,6-dihydrouracil; 5-OHC, 5-hydroxycytosine; Tg, thymine glycol; DHT, 5,6-dihydrothymine; Ih, 5-carboxamido-5-formamido-2-iminohydantoin; FapyA, 4,6-diamino-5-formamidopyrimidine; FapyG, 2,6-diamino-4-hydroxy-5-formamidopyrimidine; Gh, guanidinohydantoin; Sp, spiroiminodihydantoin; and Ur, urea residue.

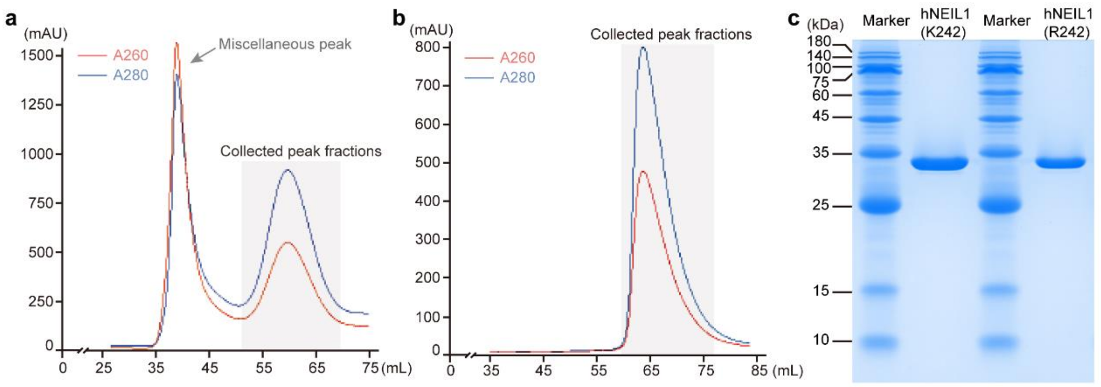

Supplementary Figure 2. Chromatographic curves and gel image for protein purification.

a, Representative chromatographic curve for the affinity purification of hNEIL1 proteins. A 5 mL HisTrap Ni-NTA column was used on an ÄKTA pure system and the recombinant hNEIL1 protein eluted at an imidazole concentration of 120 mM. **b**, Representative chromatographic curve for the gel filtration purification of hNEIL1 proteins. A Superdex 75 PG chromatography column (120 mL) was used here. **c**, Coomassie brilliant blue staining images for the purified hNEIL1 proteins. The molecular weight for hNEIL1 proteins is ~33 kDa. This experiment was repeated five times independently with consistent results.

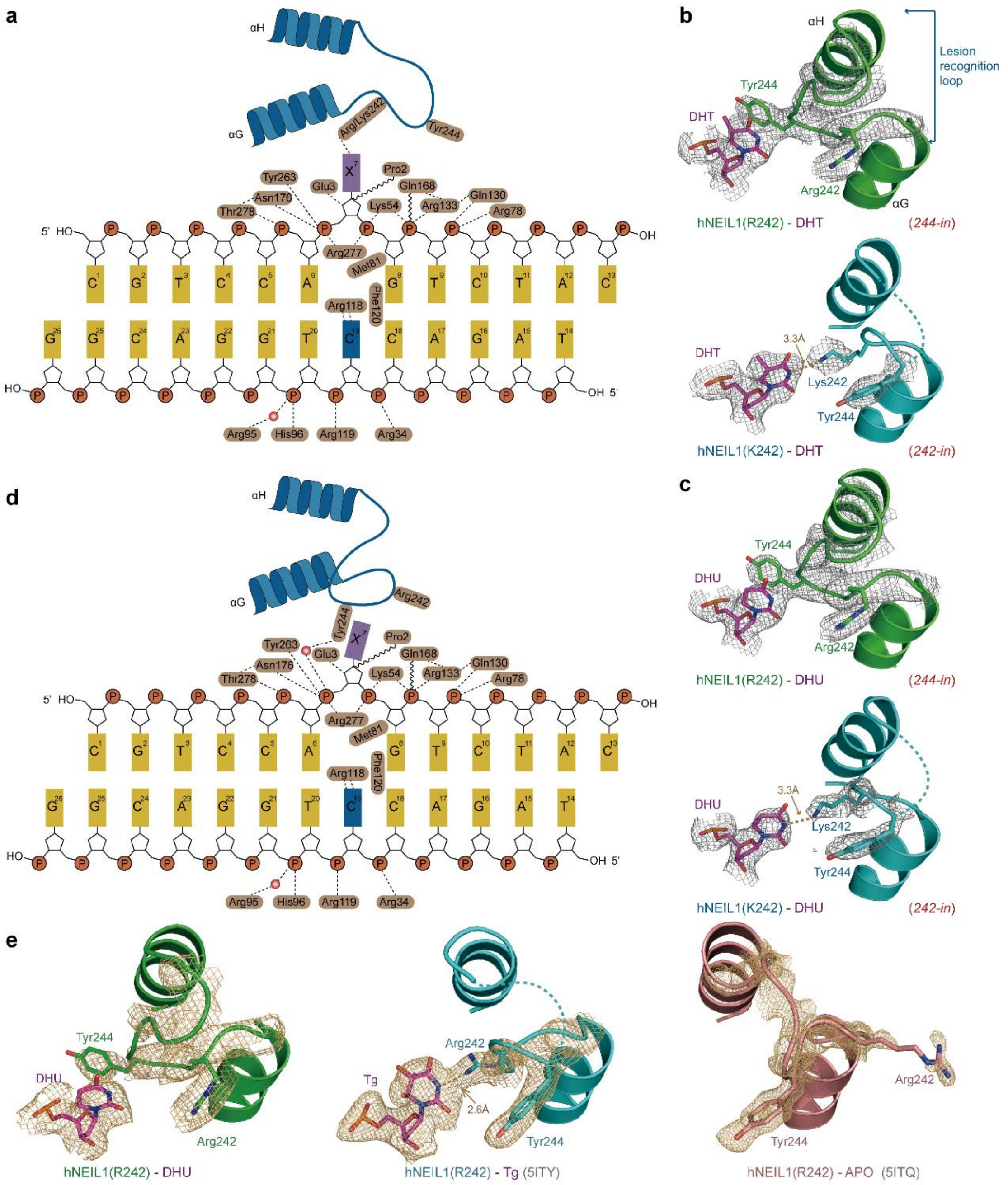

Supplementary Figure 3. Characterization of hNEIL1-DNA interactions.

a, Diagram of hNEIL1-DNA interface for *242-in* conformation. Dashed lines represent hydrogen bonds and wavy lines represent van der Waals interactions. DNA backbone phosphates, bases and amino acid residues are indicated with orange, gold and brown, respectively. Water molecules are represented by red balls. The purple and blue rectangles stand for the flipped base and the estranged cytosine, respectively. **b**, A closer view of hNEIL1(R242)-DHT structure (upper) shows that DHT base is recognized in a very different mode. Compared with hNEIL1(K242)-DHT structure (lower), hNEIL1(R242) adopts a *244-in* loop conformation, where Tyr244 stacks against the flipped base while Arg242 moves far away from DHT. The 2Fo-Fc simulated-annealing composite omit electron density map

is shown here in grey mesh and contoured at 1.0σ. **c**, The *244-in* loop conformation was also observed in the crystal structure of hNEIL1(R242) in complex with DHU. **d**, Diagram of hNEIL1-DNA interface for *244-in* conformation. **e**, Crystal structures of hNEIL1(R242) bound to DHU (left), Tg (middle) or APO (right) reveals three different conformations of the lesion recognition loop. The annealed Fo-Fc omit map is shown here in orange mesh and contoured at 2.5σ.

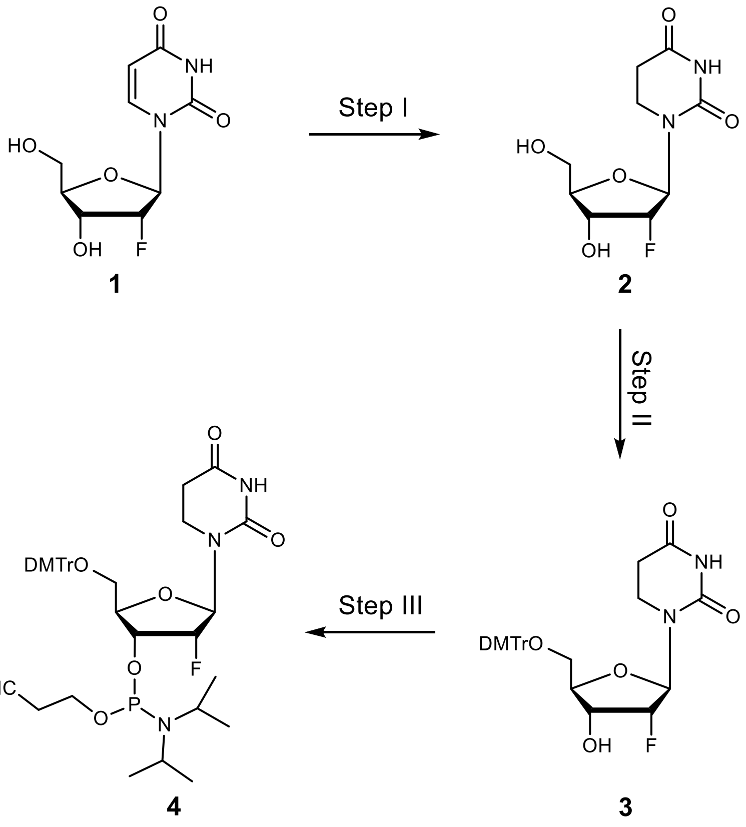

Supplementary Figure 4. The three-step synthesis route of FDHU phosphoramidite.

(1) Step I: Rh/C (5%), H_2;

(2) Step II: DMTrCl, pyridine;

(3) Step III: amidite reagent, DIPEA, CH_2Cl_2.

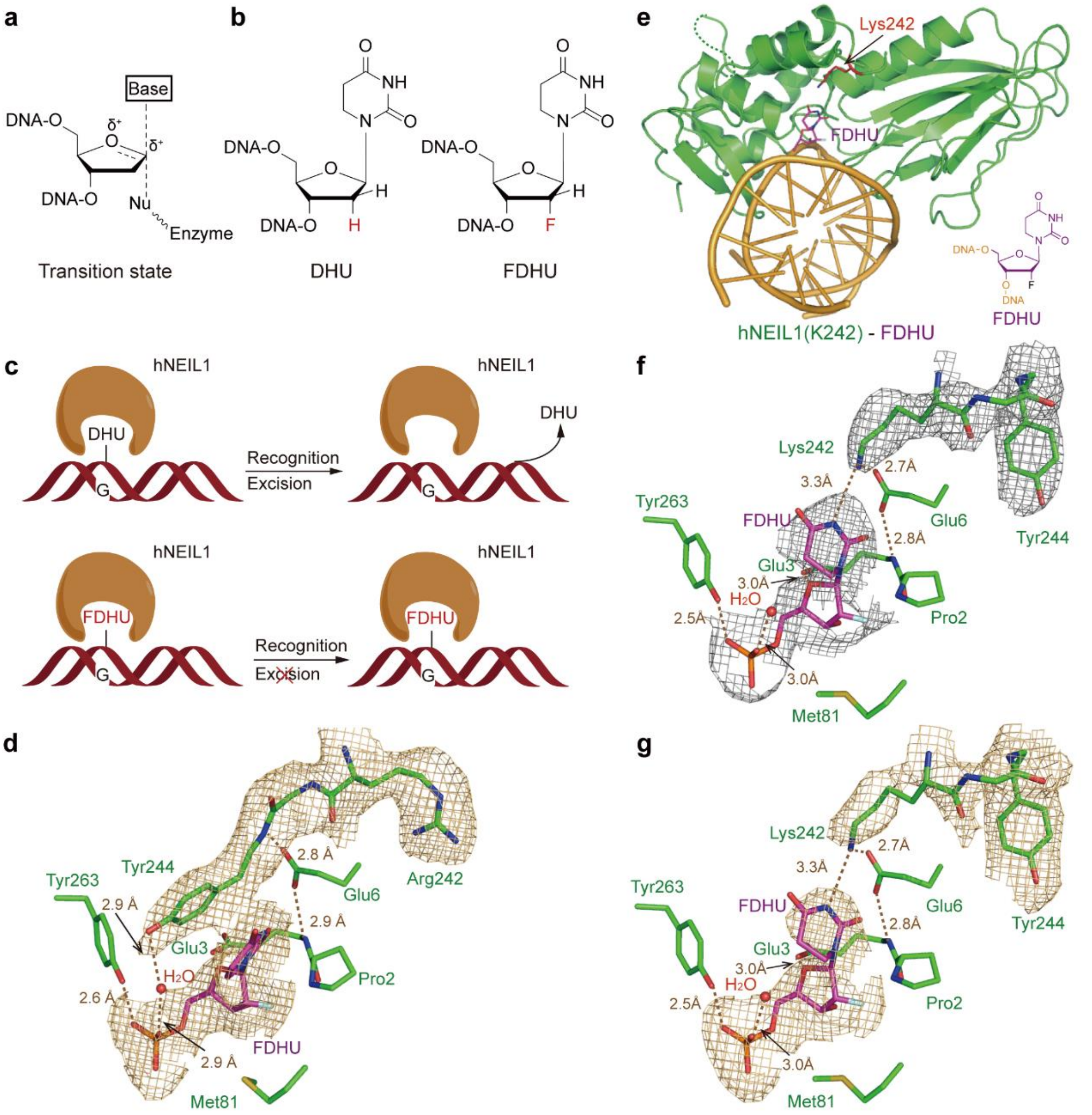

Supplementary Figure 5. Design of FDHU for the co-crystallization with hNEIL1.

a, The structure of transition state for glycosidic bond cleavage by DNA glycosylases. Significant positive charge is accumulated on the ribose ring of substrate (especially at the C1' and O1'). **b**, FDHU is a stable mimicry of DHU. Introduction of an electron-withdrawing fluorine group at C2' is expected to electronically destabilize the positive charge of transition state, therefore resulting in a decreased reaction rate. **c**, hNEIL1 can form stable complex with FDHU-containing double-strand DNA. Different from DHU which could be recognized and efficiently removed by wild-type hNEIL1, the glycosidic bond connecting FDHU and ribose ring is stabilized toward the excision reaction, thus enabling the study of interaction between hNEIL1 and damaged base via structural and biochemical approaches. **d**, Zoom-in view of the interactions between hNEIL1(R242) and FDHU. A *244-in* loop conformation can be observed here. The annealed difference Fo-Fc omit map is contoured at 2.5σ. **e**, Overall structure of hNEIL1(K242) bound to FDHU-containing DNA. The FDHU

deoxynucleotide is colored in purple, and side chain of Lysine242 is indicated with red. **f**, Zoom-in view of the interactions between hNEIL1(K242) and FDHU. The *242-in* loop conformation can be observed in the hNEIL1(K242)-FDHU structure. The brown dashed lines represent hydrogen bonds and the red ball stands for water molecule. The 2Fo-Fc simulated-annealing composite omit electron density map is shown in grey mesh and contoured at 1.0σ. **g**, Zoom-in view of the interactions between hNEIL1(K242) and FDHU. The annealed Fo-Fc omit map is shown here in orange mesh and contoured at 2.5σ.

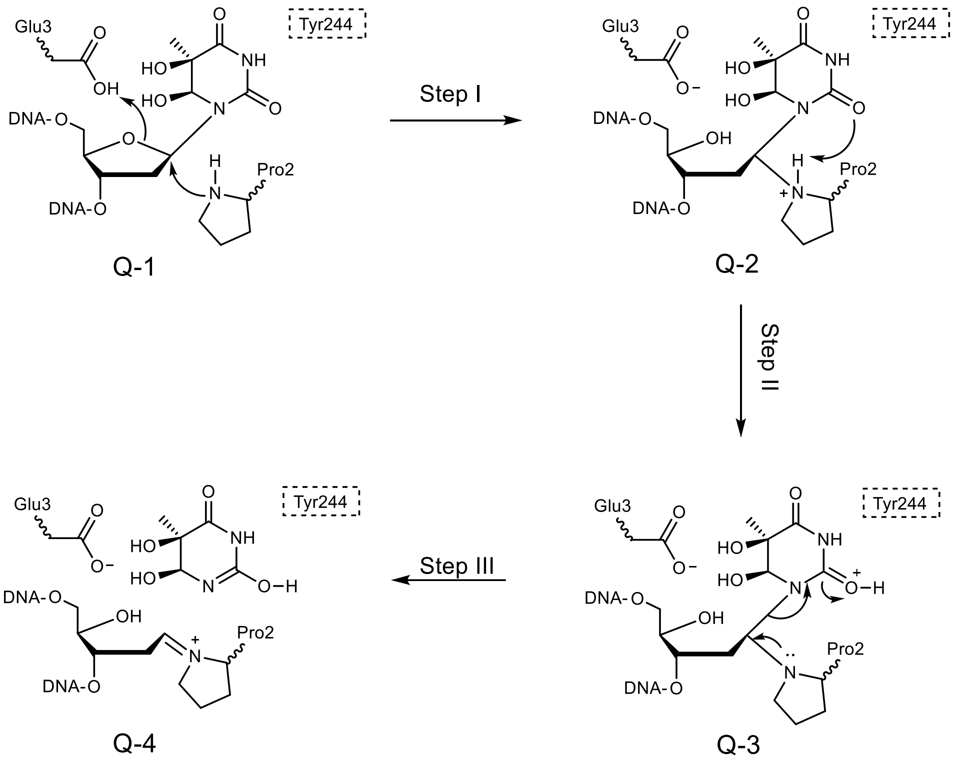

Supplementary Figure 6. Ribose-protonated catalysis pathway for *244-in* loop conformation.

The reactant (Q-1), intermediates (Q-2 and Q-3) and product (Q-4) are shown step-wisely. The reaction coordinate(s) used for QM/MM simulation of the reaction free-energy profile is:

(1) Step I: RC1 = $d(C1'_{Tg}\text{-}O4'_{Tg}) - d(C1'_{Tg}\text{-}N_{Pro2}) - d(O4'_{Tg}\text{-}H_{Glu3})$; Both Pro2 and Glu3 are in the neutral form.

(2) Step II: RC2 = $- d(O2_{Tg}\text{-}H_{Pro2})$

(3) Step III: RC3 = $d(C1'_{Tg}\text{-}N1_{Tg})$

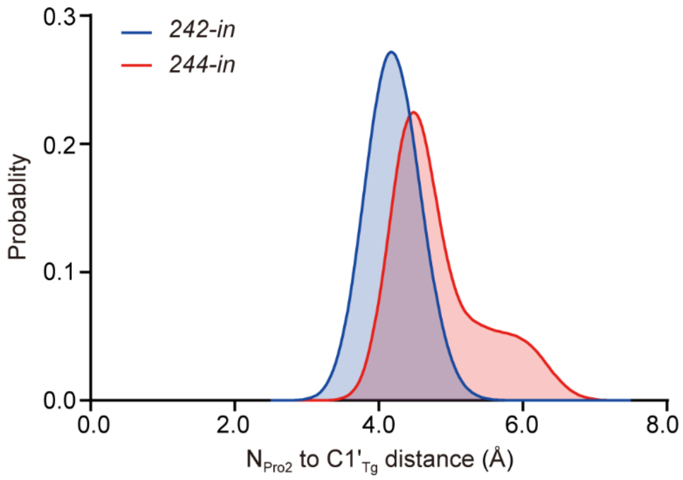

Supplementary Figure 7. The calculated distribution of attacking distance between N atom of Pro2 and C1' atom of the flipped ribose for different loop conformations. The nitrogen atom on Pro2 to the C1' atom of the flipped nucleotide is the key step that initializes the enzymatic reaction, and hence the distance between these two atoms is indicative of the ease of the reaction initialization. The force-field simulations were launched using M1 and M7 models (Supplementary Table 2), respectively.

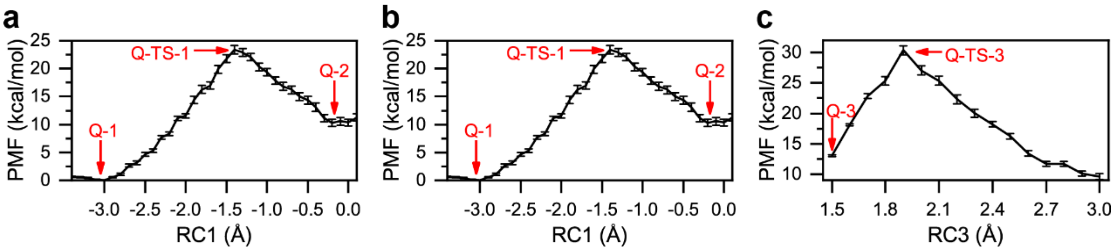

Supplementary Figure 8. QM/MM umbrella sampling of the *244-in* catalysis pathway. **a**, Potential of mean force (PMF) for "Step I" reaction described in Supplementary Fig. 6. **b**, Potential of mean force (PMF) for the "Step II" reaction described in Supplementary Fig. 6. **c**, Potential of mean force (PMF) for "Step III" reaction described in Supplementary Fig. 6. All the landmarks, including Q-1, Q-TS-1, Q-2, Q-TS-2, Q3 and Q-TS-3, are indicated by the red arrows and shown in Fig. 2d. Note that we did not further the computation of the product release step. In Fig. 2d, we assumed the final released state of *244-in* pathway (i.e., Q-4 in Fig. 2d) is identical to the *242-in* pathway (i.e., A-4 in Fig. 2d). The line plots correspond to the WHAM-estimated free energy along the reaction coordinate(s). The bars shown in the plots correspond to the Bayesian estimations of the standard errors (SEM) of the free energy profiles, which were computed through a Monte Carlo bootstrapping procedure with 100 independent bootstrapped trajectories (n = 100)[22,23].

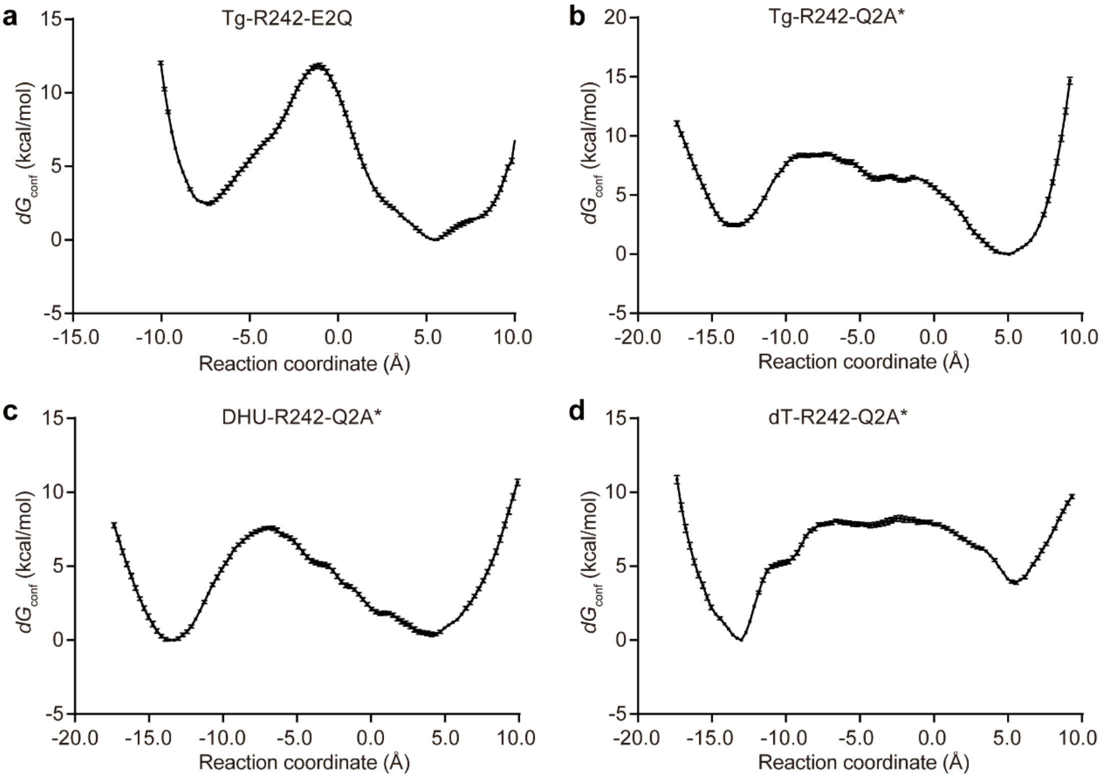

Supplementary Figure 9. Umbrella sampling of the free energy of the loop conformational transition.

a, Conformational transition from encounter state (E state) to quarantine state (Q state) for hNEIL1-Tg complex. Umbrella sampling was performed along the reaction coordinate: -0.799*d*(R242-base)-0.554*d*(R242-F85)+0.068*d*(Y244-base)+0.142*d*(Y244-F85). **b**, Conformational transition from quarantine (Q state) to pre-active (A* stands for a configuration where the loop adopts *242-in* conformation and the base is adequately flipped out, but no tautomerization events occur) for hNEIL1-Tg complex. Umbrella sampling was performed along the reaction coordinate: -0.219*d*(R242-base)+0.583*d*(R242-F85)+0.905*d*(Y244-base)-0.759*d*(Y244-F85) **c**, Conformational transition from quarantine (Q state) to pre-active for hNEIL1-DHU complex. Umbrella sampling was performed along the same reaction coordinate as in (**b**). **d**, Conformational transition from quarantine (Q state) to pre-active for hNEIL1-dT complex. Umbrella sampling was performed along the same reaction coordinate as in (**b**). The line plots correspond to the WHAM-estimated free energy along the reaction coordinate(s). The bars shown in the plots correspond to the Bayesian estimations of the standard errors (SEM) of the free energy profiles, which were computed through a Monte Carlo bootstrapping procedure with 200 independent bootstrapped trajectories (n = 200)[22,23].

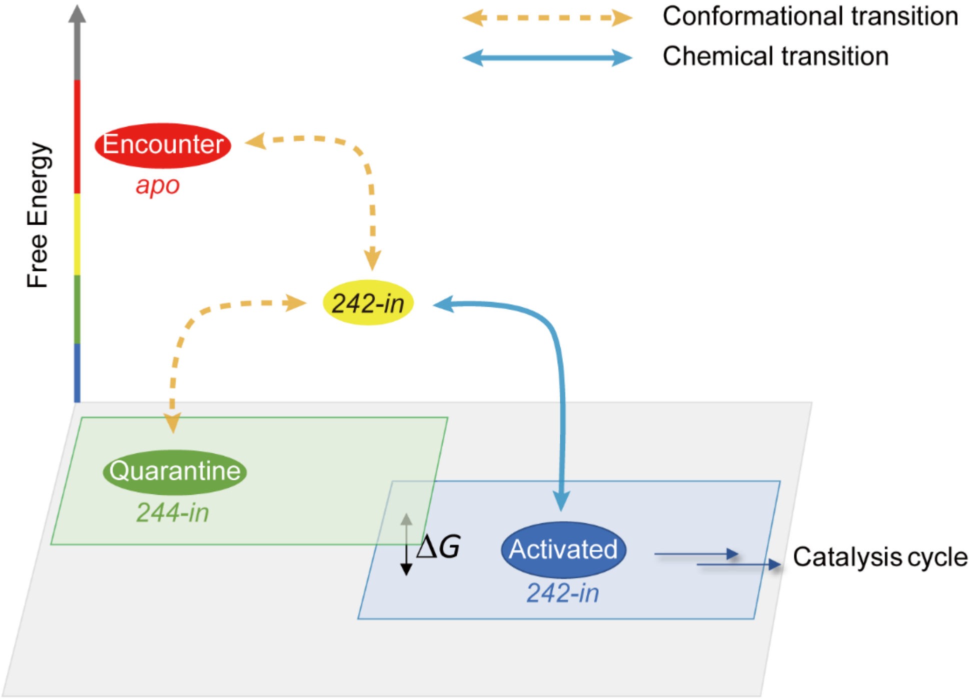

Supplementary Figure 10. A diagram illustrating the landscape of free energy distribution for different hNEIL1-DNA interaction states.

The encounter state (red) is relatively unstable and the lesion recognition loop of hNEIL1 adopts an *apo* conformation at this point. Then the loop shifts toward the DNA to form *242-in* conformation (gold) and residue 242 attempts to engage with the flipped nucleobase. Once the tautomerization-dependent recognition is established between the base and residue 242, the substrate is activated (blue) and permitted to enter the catalytic cycle for excision. Alternatively, the lesion recognition loop may also bypass the activated state and transit to the *244-in* conformation (quarantine state, green), which temporally protects the nucleobase from cleavage. The free energy difference between the quarantine state and activated state (ΔG) determines their relative dominance in equilibrium population of the protein-DNA complex. The gold dashed line represents a process involving conformational transition and the blue solid line means a chemical transition.

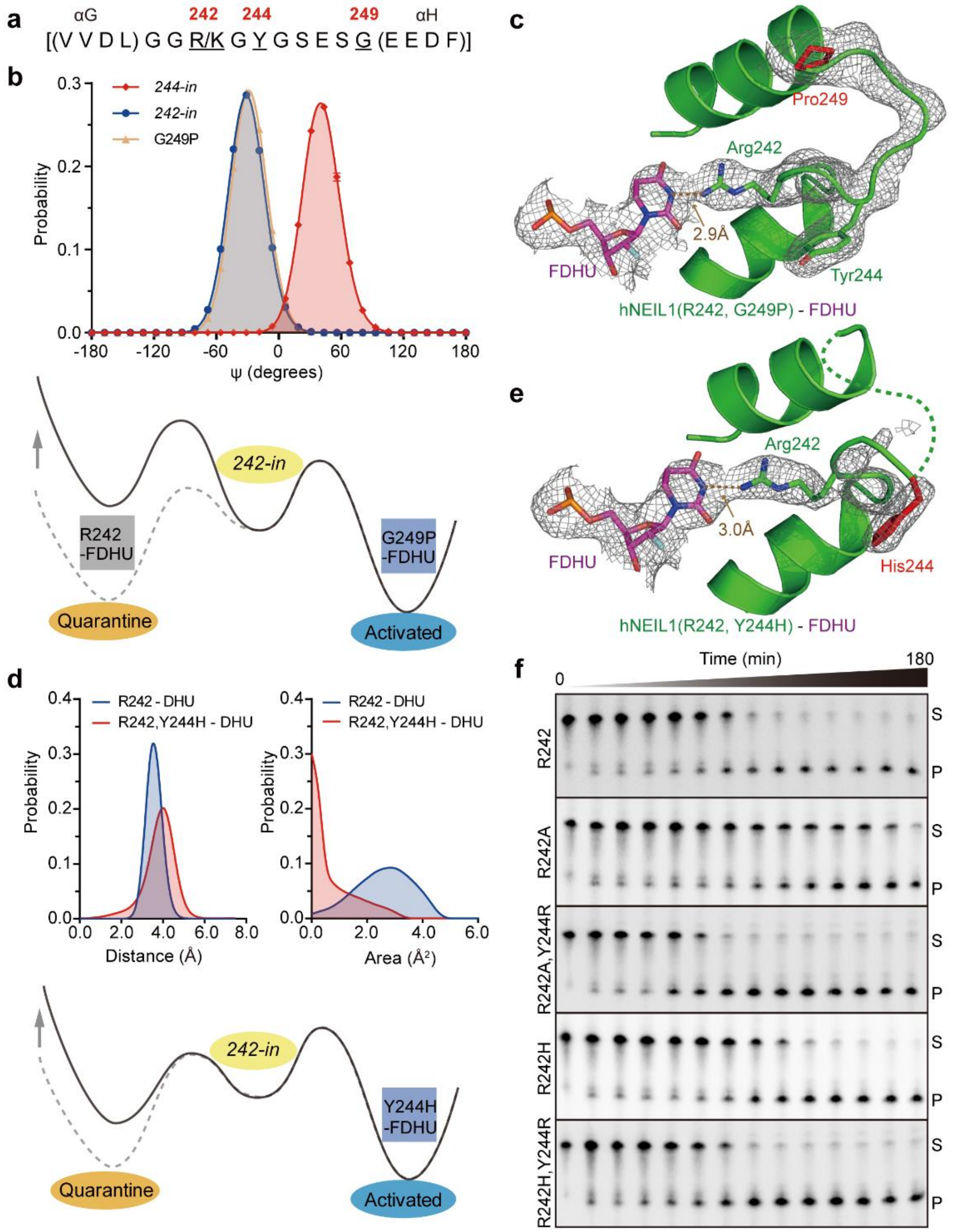

Supplementary Figure 11. Manipulation of state transition by rationally designed hNEIL1 mutants.

a, The amino acid sequence of lesion recognition loop. **b**, MD simulations of hNEIL1-DHU complex (upper) reveal that the distribution of ψ angle for residue 249 is highly consistent with that in *242-in* loop conformation, rather than *244-in* conformation, when replacing Glycine with a Proline. The solid-line curves and the associated bars correspond to the estimated mean value(s) and the standard deviations (SD) of the probability density, respectively, which were estimated by 20 blocks of the simulation trajectory (n = 20). A

diagram (lower) also illustrates a proposed free energy profile for the state transition of hNEIL1(R242, G249P) bound to FDHU-containing dsDNA. Dotted line, hNEIL1(R242)-FDHU; solid line, hNEIL1(R242, G249P)-FDHU. **c**, A closer view of hNEIL1(R242, G249P)-FDHU reveals a *242-in* conformation of lesion recognition loop with clear electron density. The 2Fo-Fc simulated-annealing composite omit electron density map is shown in grey mesh and contoured at 1.0σ. **d**, Computational simulations show the differences in the distances (upper left) and overlapping areas (upper right) between DHU base and sidechain of Tyr/His residue at position 244. The proposed free energy map (lower) for the state transition of hNEIL1(R242, Y244H) in complex with duplex DNA containing FDHU is also illustrated. **e**, The recognition of FDHU by hNEIL1(R242, Y244H) shows a similar *242-in* loop conformation as in (**c**). **f**, Representative gel images for the single-turnover kinetics study of hNEIL1 mutants on DHU (Fig. 4b in the main text). The experiment was repeated three times independently with similar results.

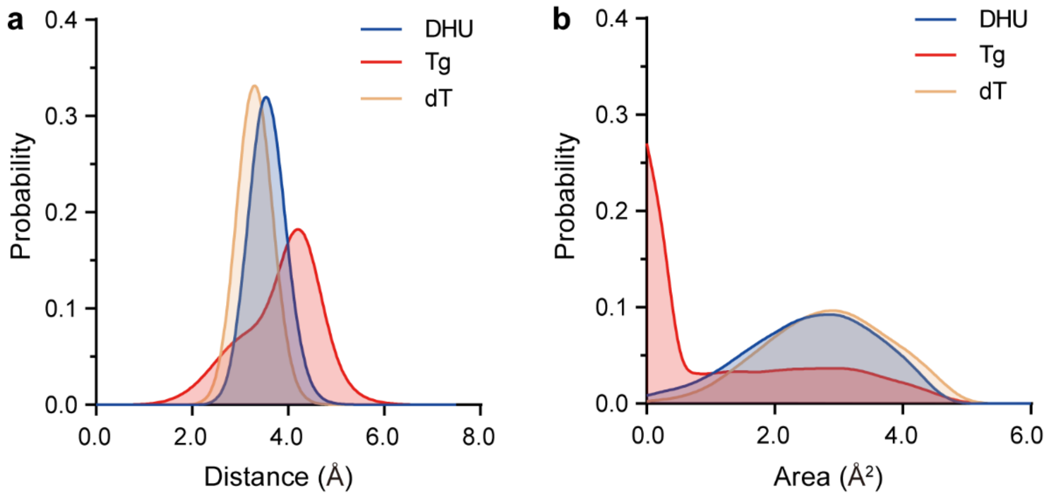

Supplementary Figure 12. The stacking features with Tyr244 for different flipped bases.

a, Computational simulations show the probability distribution of the distances between Tyr244 and flipped bases that carry different aromaticity. **b**, The overlapping areas for different bases when stacking with residue Tyr244. In both (**a**) and (**b**), the distribution for DHU, Tg and dT are colored in blue, red and sandy, respectively.

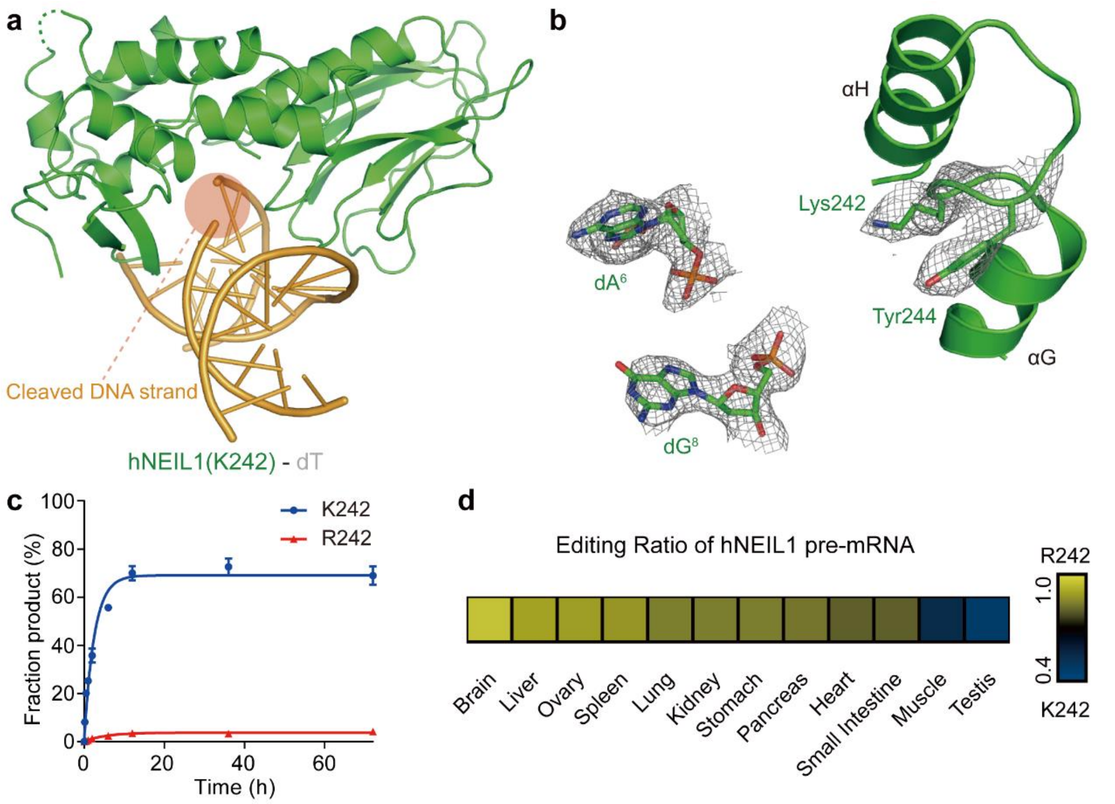

Supplementary Figure 13. The RNA editing event regulates the DNA repair accuracy and efficiency of hNEIL1.

a, The overall structure of unedited hNEIL1(K242) in complex with duplex DNA containing a cleaved T:C mismatch. The DNA and protein are colored in gold and green, respectively. A single-strand break indicated with orange background can be found in the DNA strand. **b**, Zoom-in view of hNEIL1(K242)-dT structure shows that thymine base is removed by the enzyme, leaving a single-strand break at the cleavage site with two phosphate groups at both 3' end of dA^6 and 5' end of dG^8. The lesion recognition loop of unedited hNEIL1(K242) adopts the *242-in* conformation, with Lys242 pointing to the position which should have been originally taken by the flipped base. The simulated-annealing 2Fo-Fc composite omit electron density map is shown in grey mesh and contoured at 1.0σ. **c**, The time course experiment shows the glycosylase activities of unedited hNEIL1(K242) and edited hNEIL1(R242) on double-strand DNA containing a T:C mismatch. Values represent mean ± s.e.m (n = 3). **d**, Editing ratio of hNEIL1 pre-mRNA within different human tissues. The editing level of hNEIL1 transcript shows a varied distribution within different tissues. The bioinformatics analysis is based on the existing RNA-seq data (sequence read archive: SRP039090).

Supplementary Tables

Supplementary Table 1. X-ray data collection and refinement statistics.

	hNEIL1(P2G, E3Q, K242) - OHU	hNEIL1(P2G, E3Q, R242) - OHU	hNEIL1(P2G, E3Q, K242) - *(S)*-Sp	hNEIL1(P2G, E3Q, R242) - *(S)*-Sp
**Data collection**				
Space Group	$P2_12_12_1$	$P2_12_12_1$	$P2_12_12$	$P2_12_12_1$
Cell dimensions				
a, b, c (Å)	73.56, 109.77, 169.17	73.81, 109.24, 169.40	88.76, 143.61, 71.25	73.59, 109.31, 168.24
α, β, γ (°)	90.00, 90.00, 90.00	90.00, 90.00, 90.00	90.00, 90.00, 90.00	90.00, 90.00, 90.00
Resolution (Å)	92.09~2.75 (2.80~2.75) *	91.81~2.55 (2.59~2.55)	75.26~2.91 (2.96~2.91)	91.66~2.40 (2.47~2.43)
R_{meas} (%)	15.1 (85.5)	9.3 (106.9)	8.9 (105.2)	9.1 (110.7)
R_{pim} (%)	6.0 (34.7)	3.4 (39.8)	3.7 (42.7)	4.0 (47.1)
$CC_{1/2}$	0.991 (0.837)	0.999 (0.788)	0.997 (0.885)	0.996 (0.780)
I / σI	16.55 (2.00)	26.77 (2.48)	26.20 (2.96)	24.74 (2.31)
Completeness (%)	100.0 (100.0)	99.8 (100.0)	99.3 (100.0)	99.6 (100.0)
Redundancy	6.4 (6.0)	7.2 (7.1)	5.8 (6.0)	5.3 (5.5)
**Molecular replacement**				
LLG	8036	9279	4298	7941
Z-score	48.6	52.5	55.8	45.9
Resolution (Å)	49.53~2.76	32.32~2.55	37.67~2.91	42.46~2.41
**Refinement**				
Resolution (Å)	92.09~2.76	91.81~2.55	75.51~2.90	91.66~2.40
No. unique reflections	36081	45294	20731	53542
R_{work}	0.2075	0.2096	0.1906	0.2111
R_{free}	0.2459	0.2530	0.2429	0.2540
No. atoms	7504	7576	5237	7657
Protein (aa)	2-290	2-290	2-290	2-290
DNA (bp)	1-13	1-13	1-13	1-13
Water	138	257	55	293
R.m.s. deviations				
Bond lengths (Å)	0.0094	0.0095	0.0098	0.0079
Bond angles (°)	1.6275	1.5417	1.7799	1.6609
Ramachandran plot statistics (%)				
Preferred	95.08	95.19	93.01	96.20
Allowed	4.78	4.81	6.99	3.80
Disallowed	0.14	0.00	0.00	0.00
PDB code	6LWA	6LWB	6LWC	6LWD

	hNEIL1(P2G, E3Q, K242) - Gh	hNEIL1(P2G, E3Q, R242) - Gh	hNEIL1(P2G, E3Q, K242) - DHT	hNEIL1(P2G, E3Q, R242) - DHT
**Data collection**				
Space Group	$P2_12_12$	$P2_12_12_1$	$P2_12_12_1$	$P2_12_12_1$
Cell dimensions				
a, b, c (Å)	88.60, 142.56, 71.07	73.80, 109.25, 168.25	73.59, 109.01, 168.05	74.01, 108.61, 170.14
α, β, γ (°)	90.00, 90.00, 90.00	90.00, 90.00, 90.00	90.00, 90.00, 90.00	90.00, 90.00, 90.00
Resolution (Å)	75.26~2.79 (2.84~2.79)	91.64~2.53 (2.59~2.53)	91.47~2.78 (2.83~2.78)	91.55~2.72 (2.77~2.72)
R_{meas} (%)	8.2 (109.1)	8.1 (103.4)	9.7 (68.0)	11.1 (99.4)
R_{pim} (%)	3.3 (43.3)	4.2 (53.0)	3.7 (25.7)	4.4 (38.3)
$CC_{1/2}$	0.998 (0.868)	0.999 (0.850)	0.996 (0.869)	0.996 (0.785)
I / σI	26.62 (2.16)	19.1 (2.1)	16.08 (2.00)	17.67 (2.00)
Completeness (%)	99.5 (100.0)	99.3 (94.5)	99.9 (100.0)	100.0 (100.0)
Redundancy	6.1 (6.4)	7.0 (7.0)	6.6 (6.8)	6.4 (6.6)
**Molecular replacement**				
LLG	5147	7447	8762	6832
Z-score	60.9	45.4	53.3	45.2
Resolution (Å)	37.60~2.79	29.67~2.53	49.36~2.78	49.66~2.70
**Refinement**				
Resolution (Å)	75.26~2.79	91.64~2.53	91.47~2.78	91.55~2.71
No. unique reflections	23022	46433	34944	38272
R_{work}	0.1856	0.2075	0.2004	0.2050
R_{free}	0.2433	0.2537	0.2423	0.2480
No. atoms	5292	7518	7570	7761
Protein (aa)	2-290	2-290	2-290	2-290
DNA (bp)	1-13	1-13	1-13	1-13
Water	64	291	153	312
R.m.s. deviations				
Bond lengths (Å)	0.0082	0.0096	0.0094	0.0099
Bond angles (°)	1.7006	1.7329	1.6267	1.6096
Ramachandran plot statistics (%)				
Preferred	94.39	94.76	94.98	93.65
Allowed	5.42	5.10	4.88	6.22
Disallowed	0.19	0.14	0.14	0.14
PDB code	6LWF	6LWG	6LWH	6LWI

	hNEIL1(P2G, E3Q, K242) - DHU	hNEIL1(P2G, E3Q, R242) - DHU	hNEIL1(R242) - FDHU	hNEIL1(K242) - FDHU
**Data collection**				
Space Group	$P2_12_12_1$	$P2_12_12_1$	$P2_12_12_1$	$P2_12_12_1$
Cell dimensions				
a, b, c (Å)	73.97, 108.94, 168.32	73.87, 109.10, 169.32	73.77, 108.65, 170.58	73.69, 109.25, 170.87
α, β, γ (°)	90.00, 90.00, 90.00	90.00, 90.00, 90.00	90.00, 90.00, 90.00	90.00, 90.00, 90.00
Resolution (Å)	91.47~2.82 (2.87~2.82)	91.72~2.88 (2.92~2.87)	91.64~2.55 (2.59~2.55)	92.04~2.67 (2.72~2.67)
R_{meas} (%)	11.3 (78.7)	12.8 (75.1)	9.3 (105.5)	9.2 (110.3)
R_{pim} (%)	4.5 (31.3)	5.1 (29.7)	3.7 (41.6)	4.1 (47.8)
$CC_{1/2}$	0.997 (0.814)	0.996 (0.840)	0.997 (0.707)	0.997 (0.693)
I / σI	19.47 (1.92)	16.35 (2.00)	24.35 (2.05)	23.01 (2.23)
Completeness (%)	99.9 (100.0)	99.7 (99.1)	99.8 (100.0)	99.3 (100.0)
Redundancy	6.4 (6.2)	6.2 (6.2)	6.1 (6.3)	5.0 (5.2)
**Molecular replacement**				
LLG	5991	6192	8199	7422
Z-score	42.1	44.5	46.6	47.4
Resolution (Å)	49.49~2.83	49.58~2.88	36.05~2.54	39.42~2.67
**Refinement**				
Resolution (Å)	91.46~2.83	91.72~2.88	91.64~2.54	92.04~2.67
No. unique reflections	33246	31849	46183	39901
R_{work}	0.1998	0.2010	0.2091	0.2048
R_{free}	0.2412	0.2399	0.2525	0.2582
No. atoms	7557	7672	7720	7479
Protein (aa)	2-290	2-290	2-290	2-290
DNA (bp)	1-13	1-13	1-13	1-13
Water	156	146	222	201
R.m.s. deviations				
Bond lengths (Å)	0.0099	0.0081	0.0100	0.0102
Bond angles (°)	1.6580	1.5539	1.5827	1.6375
Ramachandran plot statistics (%)				
Preferred	95.50	95.15	95.69	95.13
Allowed	4.37	4.71	4.04	4.59
Disallowed	0.14	0.13	0.27	0.28
PDB code	6LWJ	6LWK	6LWL	6LWM

	hNEIL1(R242, G249P) - FDHU	hNEIL1(R242, Y244H) - FDHU	hNEIL1(R242, Y244R) - FDHU	hNEIL1(R242) - dT
**Data collection**				
Space Group	$P2_12_12_1$	$P2_12_12_1$	$P2_12_12_1$	$P2_12_12_1$
Cell dimensions				
a, b, c (Å)	73.77, 109.99, 172.18	73.93, 109.45, 170.6	73.94, 109.64, 171.94	70.08, 109.23, 171.99
α, β, γ (°)	90.00, 90.00, 90.00	90.00, 90.00, 90.00	90.00, 90.00, 90.00	90.00, 90.00, 90.00
Resolution (Å)	92.69~2.74 (2.79~2.74)	92.13~2.48 (2.52~2.48)	92.45~2.64 (2.69~2.64)	92.21~2.88 (2.93~2.88)
R_{meas} (%)	10.3 (110.2)	8.4 (109.4)	10.1 (109.9)	11.8 (93.5)
R_{pim} (%)	4.3 (45.6)	3.4 (44.6)	4.4 (46.8)	5.6 (49.8)
$CC_{1/2}$	0.998 (0.667)	0.998 (0.707)	0.998 (0.715)	0.998 (0.490)
I / σI	21.22 (2.00)	28.30 (2.67)	20.8 (2.09)	14.86 (2.09)
Completeness (%)	99.8 (100.0)	99.7 (100.0)	99.7 (100.0)	97.7 (97.0)
Redundancy	5.5 (5.6)	5.9 (6.1)	5.0 (5.2)	4.5 (4.6)
**Molecular replacement**				
LLG	6847	8907	7397	5234
Z-score	49.0	50.6	47.2	40.5
Resolution (Å)	45.30~2.73	36.12~2.51	36.14~2.64	43.00~2.89
**Refinement**				
Resolution (Å)	92.73~2.73	92.13~2.51	92.45~2.64	92.21~2.89
No. unique reflections	37876	48248	41853	31997
R_{work}	0.1992	0.2027	0.2034	0.2372
R_{free}	0.2454	0.2428	0.2319	0.2828
No. atoms	7854	7588	7673	7521
Protein (aa)	2-290	2-290	2-290	2-290
DNA (bp)	1-13	1-13	1-13	1-13
Water	256	277	216	98
R.m.s. deviations				
Bond lengths (Å)	0.0099	0.0086	0.0093	0.0098
Bond angles (°)	1.6387	1.5409	1.6101	1.6397
Ramachandran plot statistics (%)				
Preferred	95.63	95.83	95.37	94.15
Allowed	4.23	3.89	4.63	5.71
Disallowed	0.13	0.28	0.00	0.14
PDB code	6LWN	6LWO	6LWP	6LWQ

	hNEIL1(K242) - dT
**Data collection**	
Space Group	$P2_12_12$
Cell dimensions	
a, b, c (Å)	88.99, 143.57, 71.17
α, β, γ (°)	90.00, 90.00, 90.00
Resolution (Å)	76.65~2.89 (2.94~2.89)
R_{meas} (%)	11.3 (96.1)
R_{pim} (%)	3.9 (32.6)
$CC_{1/2}$	0.997 (0.868)
I / σI	24.42 (2.05)
Completeness (%)	100.0 (100.0)
Redundancy	8.3 (8.4)
**Molecular replacement**	
LLG	4799
Z-score	60.2
Resolution (Å)	44.50~2.90
**Refinement**	
Resolution (Å)	76.65~2.90
No. unique reflections	20949
R_{work}	0.2084
R_{free}	0.2477
No. atoms	5150
Protein (aa)	2-290
DNA (bp)	1-13
Water	40
R.m.s. deviations	
Bond lengths (Å)	0.0090
Bond angles (°)	1.6229
Ramachandran plot statistics (%)	
Preferred	93.46
Allowed	6.54
Disallowed	0.00
PDB code	6LWR

* Values in parentheses are for highest-resolution shell.

Supplementary Table 2. All-atom models for hNEIL1-DNA complexes.

Model	Index	Origin
Tg-R242-242in	M1	Crystal (5ITY)
Tg-K242-242in	M2	Crystal (5ITX)
DHU-R242-244in	M3	Crystal (6LWL)
DHU-K242-242in	M4	Crystal (6LWM)
dT-R242-244in	M5	Crystal (6LWQ)
dT-K242-242in	M6	Crystal (6LWR)
Tg-R242-244in	M7	Mutated from M3
Tg-K242-244in	M8	Mutated from M3
DHU-R242-242in	M9	Mutated from crystal (6LWG) and M1
DHU-K242-244in	M10	Mutated from M3
dT-R242-242in	M11	Mutated from crystal (6LWG) and M1
dT-K242-244in	M12	Mutated from M3
DHU-(R242, G249P)-242in	M13	Crystal (6LWN)
DHU-(R242, Y244H)-244in	M14	Crystal (6LWO)
DHU-R242-apo	M15	Combine crystals (5ITQ) and (6LWL)

Supplementary Table 3. Rate constants (k_{obs}) of base removal by hNEIL1 and its mutants.

hNEIL1 mutants	k_{obs} (min^{-1}) *	
	DHU	Tg†
R242	0.090 ± 0.006	0.016 ± 0.004
R242A	0.024 ± 0.003	0.0045 ± 0.0002
R242H	0.045 ± 0.003	0.0091 ± 0.0004
R242A, Y244R	0.62 ± 0.03	0.017 ± 0.004
R242H, Y244R	0.23 ± 0.02	0.014 ± 0.003

* Rate constants in min^{-1} were determined under single-turnover conditions at 16 °C; values represent mean ± s.e.m (n = 3).

† Slow reactions rates were determined based on initial rate rather than complete fitting of the progress curve.

Supplementary Table 4. The calculated free energy value (in kcal/mol) of state transition for hNEIL1 variants in complex with different nucleobases.

Enzyme-base groups	ΔG_{conf} *	ΔG_{chem}	ΔG
hNEIL1(R242)-Tg	-2.9 (0.3)	-3.1 (0.3)	-6.0
hNEIL1(K242)-Tg		-5.4 (0.6)	-8.3
hNEIL1(R242)-DHU	0.4 (0.2)	-0.6 (0.1)	-0.2
hNEIL1(K242)-DHU		-5.1 (0.5)	-4.7
hNEIL1(R242)-dT	3.8 (0.4)	1.2 (0.2)	5.0
hNEIL1(K242)-dT		-5.0 (0.4)	-1.2

* Numbers in parentheses are statistical uncertainties.

Supplementary Table 5. The interaction states of hNEIL1 variants bound to different flipped bases observed in crystal structures.

Damaged bases	hNEIL1(K242)	hNEIL1(R242)
Tg	Activated state	Activated state
5-OHU	Activated state	Activated state
Sp1	Activated state	Activated state
Gh	Activated state	Activated state
DHT	Activated state	Quarantine state
(F)DHU	Activated state	Quarantine state
dT	Activated state	Quarantine state

Supplementary Table 6. Primers used for the plasmid construction of hNEIL1 mutants.

Primer names	Sequences
hNEIL1_R242A_F	GGCGGTGCAGGCTATGGTTCAGAATCGGGC
hNEIL1_R242A_R	ATAGCCTGCACCGCCCAGCTGAACCACTTC
hNEIL1_R242H_F	GGCGGTCATGGCTATGGTTCAGAATCGGGC
hNEIL1_R242H_R	ATAGCCATGACCGCCCAGCTGAACCACTTC
hNEIL1_R242_G249P_F	GAATCGCCGGAAGAAGATTTTGCGGCCTTCCG
hNEIL1_R242_G249P_R	ATCTTCTTCCGGCGATTCTGAACCATAGCCGC
hNEIL1_R242_Y244H_F	CGCGGCCATGGTTCAGAATCGGGCGAAGAAGAT
hNEIL1_R242_Y244H_R	TTCTGAACCATGGCCGCGACCGCCCAGCTGAAC
hNEIL1_R242_Y244R_F	CGCGGCCGTGGTTCAGAATCGGGCGAAGAAGAT
hNEIL1_R242_Y244R_R	TTCTGAACCACGGCCGCGACCGCCCAGCTGAAC
hNEIL1_R242A_Y244R_F	GCAGGCCGTGGTTCAGAATCGGGCGAAGAAGAT
hNEIL1_R242A_Y244R_R	TTCTGAACCACGGCCTGCACCGCCCAGCTGAAC
hNEIL1_R242H_Y244R_F	CATGGCCGTGGTTCAGAATCGGGCGAAGAAGAT
hNEIL1_R242H_Y244R_R	TTCTGAACCACGGCCATGACCGCCCAGCTGAAC

References

1 Schibel, A. E. *et al.* Nanopore detection of 8-oxo-7,8-dihydro-2'-deoxyguanosine in immobilized single-stranded DNA via adduct formation to the DNA damage site. *J. Am. Chem. Soc.* **132**, 17992-17995, doi:10.1021/ja109501x (2010).

2 Ferraris, D. *et al.* Design, synthesis, and pharmacological evaluation of fluorinated tetrahydrouridine derivatives as inhibitors of cytidine deaminase. *J Med Chem* **57**, 2582-2588, doi:10.1021/jm401856k (2014).

3 Onizuka, K., Yeo, J., David, S. S. & Beal, P. A. NEIL1 binding to DNA containing 2'-fluorothymidine glycol stereoisomers and the effect of editing. *Chembiochem* **13**, 1338-1348, doi:10.1002/cbic.201200139 (2012).

4 Hornak, V. *et al.* Comparison of multiple Amber force fields and development of improved protein backbone parameters. *Proteins* **65**, 712-725, doi:10.1002/prot.21123 (2006).

5 MacKerell, A. D. *et al.* All-atom empirical potential for molecular modeling and dynamics studies of proteins. *J Phys Chem B* **102**, 3586-3616, doi:10.1021/jp973084f (1998).

6 Zhu, C. *et al.* Tautomerization-dependent recognition and excision of oxidation damage in base-excision DNA repair. *Proc Natl Acad Sci U S A* **113**, 7792-7797, doi:10.1073/pnas.1604591113 (2016).

7 Anandakrishnan, R., Aguilar, B. & Onufriev, A. V. H++ 3.0: automating pK prediction and the preparation of biomolecular structures for atomistic molecular modeling and simulations. *Nucleic Acids Res* **40**, W537-541, doi:10.1093/nar/gks375 (2012).

8 Ryckaert, J.-P., Ciccotti, G. & Berendsen, H. J. C. Numerical integration of the cartesian equations of motion of a system with constraints: molecular dynamics of n-alkanes. *Journal of Computational Physics* **23**, 327-341, doi:10.1016/0021-9991(77)90098-5 (1977).

9 Sadeghian, K. *et al.* Ribose-protonated DNA base excision repair: a combined theoretical and experimental study. *Angew Chem Int Ed Engl* **53**, 10044-10048, doi:10.1002/anie.201403334 (2014).

10 de, M. S. G., Walker, R. C., Elstner, M., Case, D. A. & Roitberg, A. E. Implementation of the SCC-DFTB method for hybrid QM/MM simulations within the amber molecular dynamics package. *J Phys Chem A* **111**, 5655-5664, doi:10.1021/jp070071l (2007).

11 Elstner, M. *et al.* Self-consistent-charge density-functional tight-binding method for simulations of complex materials properties. *Physical Review B* **58**, 7260-7268, doi:DOI 10.1103/PhysRevB.58.7260 (1998).

12 Kruger, T., Elstner, M., Schiffels, P. & Frauenheim, T. Validation of the density-functional based tight-binding approximation method for the calculation of reaction energies and other data. *J Chem Phys* **122**, 114110, doi:10.1063/1.1871913 (2005).

13 Torrie, G. M. & Valleau, J. P. Nonphysical sampling distributions in Monte Carlo free-energy estimation: Umbrella sampling. *Journal of Computational Physics* **23**, 187-199, doi:https://doi.org/10.1016/0021-9991(77)90121-8 (1977).

14 Kumar, S., Rosenberg, J. M., Bouzida, D., Swendsen, R. H. & Kollman, P. A. Multidimensional Free-Energy Calculations Using the Weighted Histogram Analysis Method. *J Comput Chem* **16**, 1339-1350, doi:DOI 10.1002/jcc.540161104 (1995).

15 Ferguson, A. L., Panagiotopoulos, A. Z., Kevrekidis, I. G. & Debenedetti, P. G.

Nonlinear dimensionality reduction in molecular simulation: The diffusion map approach. *Chemical Physics Letters* **509**, 1-11, doi:10.1016/j.cplett.2011.04.066 (2011).
16 Rohrdanz, M. A., Zheng, W., Maggioni, M. & Clementi, C. Determination of reaction coordinates via locally scaled diffusion map. *J Chem Phys* **134**, 124116, doi:10.1063/1.3569857 (2011).
17 Hruska, E., Abella, J. R., Nuske, F., Kavraki, L. E. & Clementi, C. Quantitative comparison of adaptive sampling methods for protein dynamics. *J Chem Phys* **149**, 244119, doi:10.1063/1.5053582 (2018).
18 Zhang, J. *et al.* Deep Representation Learning for Complex Free-Energy Landscapes. *J Phys Chem Lett* **10**, 5571-5576, doi:10.1021/acs.jpclett.9b02012 (2019).
19 Pedregosa, F. *et al.* Scikit-learn: Machine Learning in Python. *J Mach Learn Res* **12**, 2825-2830 (2011).
20 Mendels, D., Piccini, G. & Parrinello, M. Collective Variables from Local Fluctuations. *J Phys Chem Lett* **9**, 2776-2781, doi:10.1021/acs.jpclett.8b00733 (2018).
21 Tribello, G. A., Bonomi, M., Branduardi, D., Camilloni, C. & Bussi, G. PLUMED 2: New feathers for an old bird. *Comput Phys Commun* **185**, 604-613, doi:10.1016/j.cpc.2013.09.018 (2014).
22 Efron, B. & Tibshirani, R. J. *An introduction to the bootstrap*. (Chapman & Hall, 1993).
23 Hub, J. S., de Groot, B. L. & van der Spoel, D. g_wham—A Free Weighted Histogram Analysis Implementation Including Robust Error and Autocorrelation Estimates. *Journal of Chemical Theory and Computation* **6**, 3713-3720, doi:10.1021/ct100494z (2010).
