# Supplementary material for: DNA repair glycosylase hNEIL1 triages damaged bases via competing interaction modes
Source: Nat Commun. 2021 Jul 5;12:4108. doi: 10.1038/s41467-021-24431-y (PMC8257757; doi:10.1038/s41467-021-24431-y)
Supplement: Supplementary file 2 — Reporting Summary [file 41467_2021_24431_MOESM2_ESM.pdf]

Corresponding author(s): Chengqi Yi
Last updated by author(s): Jun 8, 2021

# Reporting Summary

Nature Research wishes to improve the reproducibility of the work that we publish. This form provides structure for consistency and transparency in reporting. For further information on Nature Research policies, see our Editorial Policies and the Editorial Policy Checklist.

## Statistics

For all statistical analyses, confirm that the following items are present in the figure legend, table legend, main text, or Methods section.

| n/a | Confirmed | |
|---|---|---|
| ☐ | ☒ | The exact sample size (*n*) for each experimental group/condition, given as a discrete number and unit of measurement |
| ☐ | ☒ | A statement on whether measurements were taken from distinct samples or whether the same sample was measured repeatedly |
| ☒ | ☐ | The statistical test(s) used AND whether they are one- or two-sided<br>*Only common tests should be described solely by name; describe more complex techniques in the Methods section.* |
| ☒ | ☐ | A description of all covariates tested |
| ☒ | ☐ | A description of any assumptions or corrections, such as tests of normality and adjustment for multiple comparisons |
| ☐ | ☒ | A full description of the statistical parameters including central tendency (e.g. means) or other basic estimates (e.g. regression coefficient) AND variation (e.g. standard deviation) or associated estimates of uncertainty (e.g. confidence intervals) |
| ☒ | ☐ | For null hypothesis testing, the test statistic (e.g. *F*, *t*, *r*) with confidence intervals, effect sizes, degrees of freedom and *P* value noted<br>*Give P values as exact values whenever suitable.* |
| ☒ | ☐ | For Bayesian analysis, information on the choice of priors and Markov chain Monte Carlo settings |
| ☒ | ☐ | For hierarchical and complex designs, identification of the appropriate level for tests and full reporting of outcomes |
| ☒ | ☐ | Estimates of effect sizes (e.g. Cohen's *d*, Pearson's *r*), indicating how they were calculated |

*Our web collection on statistics for biologists contains articles on many of the points above.*

## Software and code

Policy information about availability of computer code

| | |
|---|---|
| Data collection | The raw data were processed with HKL-2000/3000 and phased by molecular replacement with Phaser (version 2.8.2). |
| Data analysis | The initial models were built in COOT (version 0.8.7) and refined with the program REFMAC5 (version 5.8.0158) from CCP4 suite (version 7.0.073). Simulated-annealing composite omit electron density map of corresponding structures were generated by Phenix (version 1.19.1) and molecular graphic figures were created with PyMOL (version 1.8.2.3).<br>The gel images were analyzed in Image Lab (version 4.0) and the reaction curves for rate constant calculation were fitted in GraphPad Prism (version 7.00).<br>All the molecular dynamics simulations were conducted on AMBER18 package. The protonation states of the initial structures were checked via H++ web server (version 3.2.2).<br>The open-sourced libraries Shapely (version 1.7.0), PyClipper (version 1.1.0) and program WHAM (version 2.0.5) were used to analyze the simulation data. |

For manuscripts utilizing custom algorithms or software that are central to the research but not yet described in published literature, software must be made available to editors and reviewers. We strongly encourage code deposition in a community repository (e.g. GitHub). See the Nature Research guidelines for submitting code & software for further information.

## Data

Policy information about availability of data

All manuscripts must include a data availability statement. This statement should provide the following information, where applicable:

- Accession codes, unique identifiers, or web links for publicly available datasets
- A list of figures that have associated raw data
- A description of any restrictions on data availability

Atomic coordinates and structure factors have been deposited in the Protein Data Bank under accession codes 6LWA, 6LWB, 6LWC, 6LWD, 6LWF, 6LWG, 6LWH, 6LWI, 6LWJ, 6LWK, 6LWL, 6LWM, 6LWN, 6LWO, 6LWP, 6LWQ and 6LWR.
Previously reported hNEIL1(R242)-Tg structure and hNEIL1(R242)-APO structure are available from the PDB under accession ID 5ITY and 5ITQ, respectively.
The RNA-seq data that support the findings of this study are available in the NCBI Sequence Read Archive under accession number SRP039090.

# Field-specific reporting

Please select the one below that is the best fit for your research. If you are not sure, read the appropriate sections before making your selection.

☒ Life sciences ☐ Behavioural & social sciences ☐ Ecological, evolutionary & environmental sciences

For a reference copy of the document with all sections, see nature.com/documents/nr-reporting-summary-flat.pdf

# Life sciences study design

All studies must disclose on these points even when the disclosure is negative.

| | |
|---|---|
| Sample size | No sample size calculations were made because this is not a population study. Sample sizes for structure determination and biochemical assays are consistent with what has been previously published in the literature (Zhu et al, PNAS 2016). |
| Data exclusions | No data were excluded from analysis. |
| Replication | All biochemical experiments were independently reproduced for three times. All attempts at replication were successful. |
| Randomization | Randomization was not performed in this paper because no animal or human subjects are involved. |
| Blinding | Blinding was not performed because it would be impossible to blind any component of the data collected. No human judgment was involved in generating the data and hence blinding is not required for this study . |

# Reporting for specific materials, systems and methods

We require information from authors about some types of materials, experimental systems and methods used in many studies. Here, indicate whether each material, system or method listed is relevant to your study. If you are not sure if a list item applies to your research, read the appropriate section before selecting a response.

## Materials & experimental systems

| n/a | Involved in the study | |
|---|---|---|
| ☒ | ☐ | Antibodies |
| ☒ | ☐ | Eukaryotic cell lines |
| ☒ | ☐ | Palaeontology and archaeology |
| ☒ | ☐ | Animals and other organisms |
| ☒ | ☐ | Human research participants |
| ☒ | ☐ | Clinical data |
| ☒ | ☐ | Dual use research of concern |

## Methods

| n/a | Involved in the study | |
|---|---|---|
| ☒ | ☐ | ChIP-seq |
| ☒ | ☐ | Flow cytometry |
| ☒ | ☐ | MRI-based neuroimaging |
